# Nanoscale water–polymer interactions tune macroscopic diffusivity of water in aqueous poly(ethylene oxide) solutions†

**DOI:** 10.1039/d3sc05377f

**Published:** 2024-01-15

**Authors:** Joshua D. Moon, Thomas R. Webber, Dennis Robinson Brown, Peter M. Richardson, Thomas M. Casey, Rachel A. Segalman, M. Scott Shell, Songi Han

**Affiliations:** a Materials Department, University of California Santa Barbara California 93106 USA; b Department of Chemical Engineering, University of California Santa Barbara California 93106 USA songi.han@northwestern.edu; c Department of Chemistry and Biochemistry, University of California Santa Barbara California 93106 USA

## Abstract

The separation and anti-fouling performance of water purification membranes is governed by both macroscopic and molecular-scale water properties near polymer surfaces. However, even for poly(ethylene oxide) (PEO) – ubiquitously used in membrane materials – there is little understanding of whether or how the molecular structure of water near PEO surfaces affects macroscopic water diffusion. Here, we probe both time-averaged bulk and local water dynamics in dilute and concentrated PEO solutions using a unique combination of experimental and simulation tools. Pulsed-Field Gradient NMR and Overhauser Dynamic Nuclear Polarization (ODNP) capture water dynamics across micrometer length scales in sub-seconds to sub-nanometers in tens of picoseconds, respectively. We find that classical models, such as the Stokes–Einstein and Mackie–Meares relations, cannot capture water diffusion across a wide range of PEO concentrations, but that free volume theory can. Our study shows that PEO concentration affects macroscopic water diffusion by enhancing the water structure and altering free volume. ODNP experiments reveal that water diffusivity near PEO is slower than in the bulk in dilute solutions, previously not recognized by macroscopic transport measurements, but the two populations converge above the polymer overlap concentration. Molecular dynamics simulations reveal that the reduction in water diffusivity occurs with enhanced tetrahedral structuring near PEO. Broadly, we find that PEO does not simply behave like a physical obstruction but directly modifies water's structural and dynamic properties. Thus, even in simple PEO solutions, molecular scale structuring and the impact of polymer interfaces is essential to capturing water diffusion, an observation with important implications for water transport through structurally complex membrane materials.

## Abstract 2

PEO restructures water near the polymer, reducing free volume and slowing local water.

## Graphical abstract

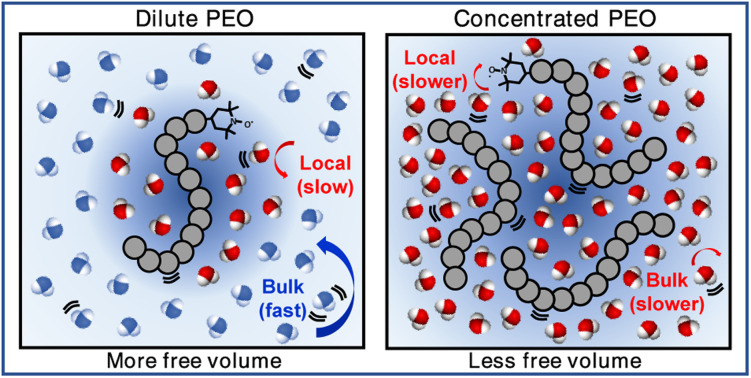

## Introduction

Polymer membranes represent a crucial technology that has enabled energy-efficient water treatment and desalination over the last few decades.^1^ At the most basic level, membranes operate by selectively permeating water faster than other solutes such as ions or organic molecules. The permeability and selectivity of water treatment membranes, which are key metrics for separation performance, are largely mediated by the dynamic and thermodynamic properties of water within a polymer membrane material.^2–6^ In porous filtration membranes, morphological effects such as pore structure and tortuosity have been shown to control transport at a macroscopic scale,^7,8^ while for non-porous membranes (*e.g.*, desalination membranes), macroscopic water transport has largely been correlated with molecular-scale geometric effects in hydrated polymer membranes, invoking terminology such as molecular obstructions or free volume.^9–12^

It is also well-established that the dynamic behavior of water in polymer solutions is necessarily mediated by interactions between water and the molecular surfaces of polymer chains.^13–18^ Despite the importance of these surface interactions, the behavior of water near polymer surfaces in hydrated membranes at the molecular level is rarely studied and poorly understood. In this study, we investigate dynamic and structural water properties in the bulk and near the molecular surfaces of poly(ethylene oxide) (PEO) in solution. We chose PEO because it is fully miscible with water and has a relatively simple repeat unit structure. PEO is also among the most widely used heuristic design elements to impart hydrophilic behavior to materials used in antifouling membrane coatings, consumer products, and biomedicine.^19–23^ Despite its simple chemical structure, PEO exhibits complex behavior in water such as weakly helical backbone conformations^24,25^ and eutectic formations over a wide range of water contents.^26^ Several existing studies of PEO and water have focused on the bulk thermodynamic phase behavior of aqueous PEO *via* differential scanning calorimetry^26–28^ or the rotational dynamics of water in PEO measured with dielectric spectroscopy.^29–33^ Fewer studies have isolated the unique effects of the hydration layer local to the PEO chain, although careful application of terahertz spectroscopy can detect differences in bound water populations relative to bulk water signatures^18,34^ and quasi-elastic neutron scattering has also been employed to sense changes in hydration layer water dynamics in PEO.^15,16,18,35–37^ Presently, there are still considerable gaps in the literature regarding the structure and dynamics of water near PEO surfaces and the choice for the most appropriate models to use to describe water diffusion in PEO solutions.

**Fig. 1:**
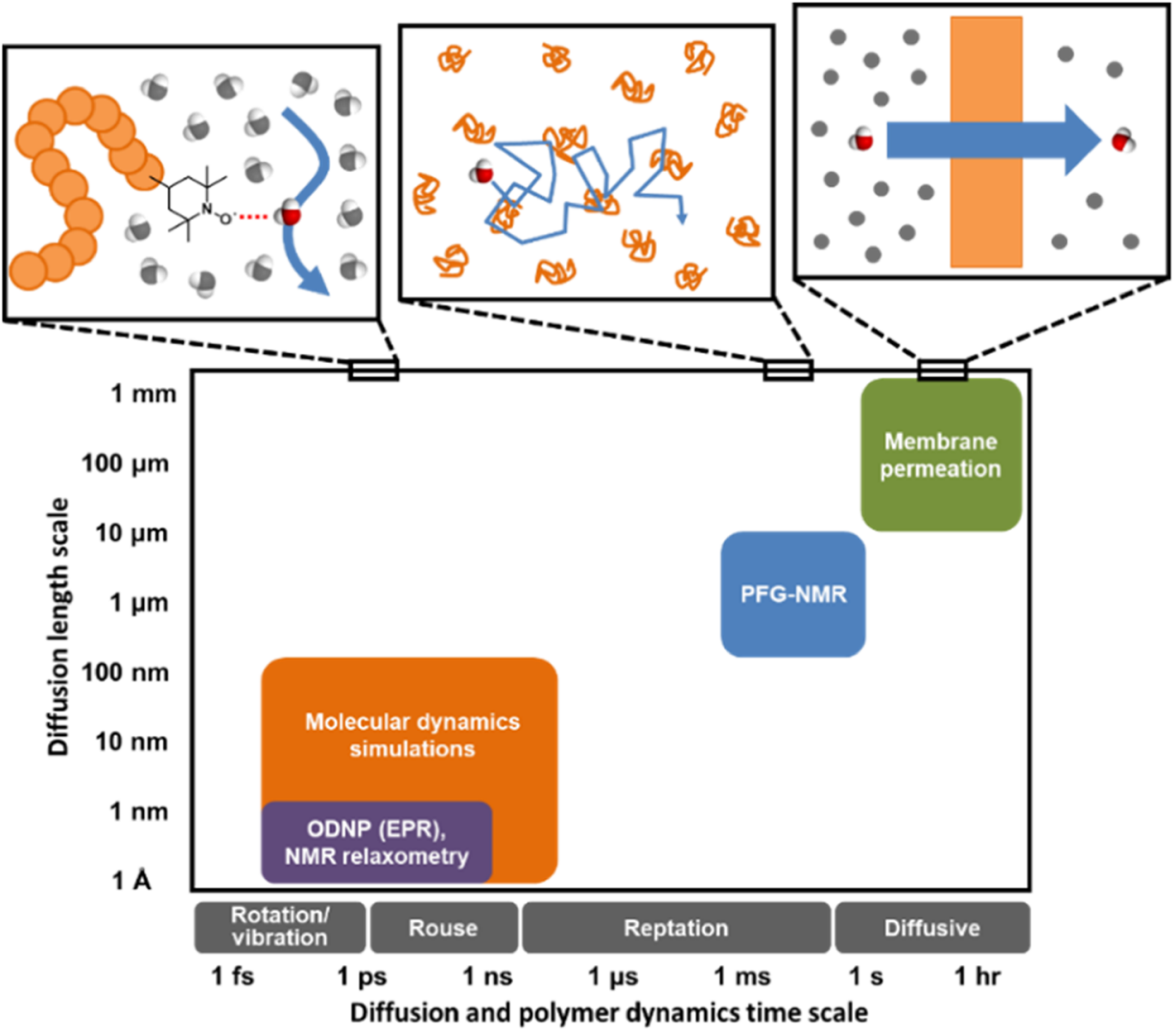
Distinct experimental and computational techniques probe small molecule diffusion processes occurring over vastly different length and time scales.^43^ Techniques such as MD simulations and ODNP can uncover molecular-scale dynamic behavior of small molecules in polymers or polymer solutions while tools like PFG-NMR and membrane permeation experiments reveal macroscopic transport phenomena.

Understanding water dynamics in PEO solutions at both the macroscale and molecular level is crucial to understanding how membrane surface chemistry and molecular interfaces affect water transport. PEO is not only a critically important model polymer, but its miscibility with water across the full concentration range from dilute to pure polymer liquid permits the study of bulk and surface water populations in contact with PEO. This study utilizes Pulsed-Field Gradient (PFG) NMR and Overhauser Dynamic Nuclear Polarization (ODNP) to study the diffusivity of water interacting with PEO in aqueous solutions. ODNP is a dual NMR and EPR technique that selectively measures water dynamics near polymer surfaces by labeling them with an electron spin probe so that the ODNP effect exclusively originates from water interacting within approximately 1 nm of the polymer surface. PFG-NMR and ODNP are highly complementary tools: respectively, they probe water dynamics at length scales from micrometers to sub-nanometers and time scales from sub-seconds to tens of picoseconds (*cf.*, Fig. 1). Molecular Dynamics (MD) simulations provide further insight into the structure and dynamics of water near PEO chains and in the bulk.

Previously employed models for transport in hydrated membranes have largely focused on the macroscopic effects of^1^ geometrical obstruction by polymer chains on diffusion,^2^ hydrodynamics due to drag arising from hard sphere collisions in a viscous medium (*e.g.*, the hydrodynamic Stokes–Einstein model), or^3^ free volume configurations.^10,12,38,39^ In contrast, studies that have investigated water diffusion near biopolymer surfaces, such as proteins or DNA, have largely focused on molecular scale dynamics rather than macroscopic transport phenomena.^40–42^ Currently, few studies, if any, have sought to directly reconcile bulk, macroscopic diffusion with the impact of molecular surface interactions and geometries, which are only indirectly treated by or are excluded from the above mentioned analytical models. Specifically, we ask under what circumstances do surface effects matter for bulk water diffusion in PEO solutions, and which macroscopic transport models adequately account for these effects across the entire concentration range from bulk water, with dilute PEO chains as solutes, to a nearly pure PEO liquid with small amounts of water interacting with the chains?

In this study, we demonstrate that structural ordering of water near PEO in solution strongly governs the diffusion of water local to the PEO chain and in the bulk. We also show that free volume theory, which implicitly accounts for molecular structural details of the solvent–polymer interactions, captures the polymer concentration effect on time-averaged water diffusivities in dilute to highly concentrated solutions more accurately than hydrodynamic or obstruction-based models. The surface effects at the polymer–water interface could be a valuable lever for controlling water dynamics near complex surfaces, such as those found in industrial polymer membranes.

## Results and discussion

### Time-averaged water self-diffusion behavior from PFG-NMR

We first investigate water's macroscale self-diffusion behavior in bulk polymer solutions. Specifically, we measure water self-diffusion coefficients in aqueous solution of PEO oligomers (MW = 550 g mol^−1^) at 21 °C as a function of PEO concentration using PFG-NMR, which captures “time-averaged” water dynamic behavior over a timeframe of milliseconds to seconds, and diffusion length scales on the order of micrometers, thus effectively probing the average mobility of water molecules across many local molecular environments. We chose solutions spanning the entire concentration range of 0 to nearly 100 wt% PEO to investigate both dilute and concentrated solution behavior below and above the overlap concentration of PEO chains. For 550 g mol^−1^ PEO oligomers in aqueous solution, this overlap concentration is 46.4 wt% and represents the point above which polymer coil volumes begin to touch, marking the transition from dilute to semi-dilute solutions.^24,44,45^ We calculate the overlap concentration using experimentally measured chain conformations, see ESI† for more details.

**Fig. 2:**
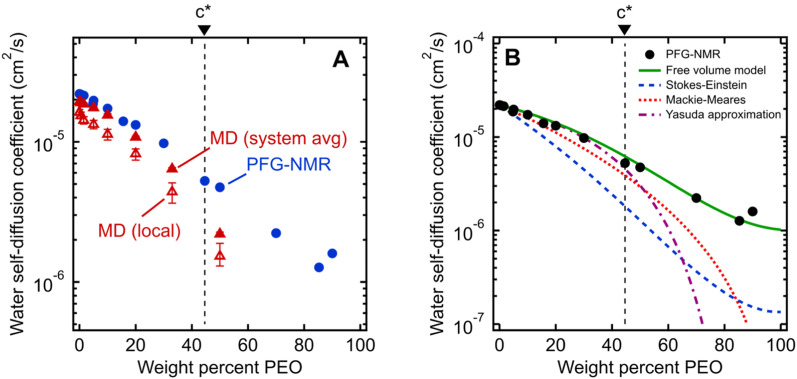
(A) Comparison of water self-diffusion coefficients in aqueous PEO solutions at room temperature (21 °C) from PFG-NMR (filled blue circles) with system-average water diffusivities (*D*_H_2_O_, filled red triangles) and local water diffusivities (*D*_local_, unfilled red triangles) from MD simulations. (B) Comparison of PFG-NMR water diffusivities in aqueous PEO solutions (black circles) with model fits for Stokes–Einstein (blue dashed curve), Mackie–Meares (red dotted curve), free volume theory (green solid curve), and Yasuda's free volume approximation (purple dashed/dotted curve). PEO overlap concentration (c*) is marked in both figures by a dashed black line (see ESI† for c* derivation).

Fig. 2A shows water self-diffusion coefficients obtained from PFG-NMR measurements as a function of PEO concentration. Water self-diffusivities decrease exponentially with increasing concentration from 2.2 × 10^−5^ cm^2^ s^−1^ for 0 wt% PEO (pure water) to 1.6 × 10^−6^ cm^2^ s^−1^ for 90 wt% PEO solutions, consistent with previous studies, although many prior efforts limited their investigations to relatively dilute PEO solutions.^46–50^ Notably, there is no evident change in the exponential scaling of diffusion with concentration for solutions above *versus* below the PEO overlap concentration; rather, a single scaling is observed across the entire concentration interval of 0 to 90 wt% PEO.

### Nanoscale water dynamics from MD simulations

Equilibrium water self-diffusivities are also calculated using Molecular Dynamics (MD) simulations for PEO solutions at the same temperature and concentrations ranging up to 50 wt% PEO. Unlike macroscale PFG-NMR measurements, MD simulations probe water dynamics over tens of picoseconds with characteristic distances on the order of nanometers. Solutions with PEO concentrations higher than 50 wt% are difficult to well-equilibrate due to the long relaxation time scales of polymer-rich systems. We calculate both the equilibrium self-diffusivities for the system-average waters, *D*_H_2_O_ (*i.e.*, all waters in the simulation box), and for the hydration layer waters, *D*_local_ (*i.e.*, waters within 8 Å of labeled chain ends), from the slope of mean squared displacement (MSD) of system-averaged and hydration layer water oxygens, respectively.

Fig. 2A shows MD-derived system-average water diffusivities (*D*_H_2_O_) as a function of PEO concentration. System-averaged water dynamics decrease exponentially with increasing PEO concentration, as previously observed in the literature,^17^ with a similar scaling to PFG-NMR diffusivities. Despite the substantial difference in length and timescales probed by the two techniques, computed values of *D*_H_2_O_ exhibit nearly quantitative agreement with PFG-NMR self-diffusivities in dilute solutions at concentrations as high as 20 wt%, with slightly slower translational dynamics predicted by MD for 33 and 50 wt% solutions. Both PFG-NMR and system-average MD capture the collective behavior of all water molecules in a solution and do not distinguish between “bulk” and hydration layer (*i.e.*, near PEO) water dynamics. *D*_local_ also exhibits the same general trend with PEO concentration as *D*_H_2_O_ but with 20 to 30% slower translational diffusion (*cf.*, Fig. 2A). This observation indicates hydration layer water molecules exhibit somewhat slower dynamics than those in the bulk solution.

### Analytical models of water diffusion in PEO solutions

#### Stokes–Einstein hydrodynamic model

To evaluate our hypothesis that molecular interactions strongly influence bulk water dynamics at the macroscale in PEO solutions, we examine the degree to which theoretical models with different levels of molecular detail can fully describe water diffusion behavior in both dilute and concentrated solutions. We first compare PFG-NMR time-averaged water self-diffusivities with the classic Stokes–Einstein equation to identify whether a purely continuum-hydrodynamic model that neglects molecular details can effectively capture the details of water diffusion behavior in PEO oligomer solutions. The Stokes–Einstein equation predicts1
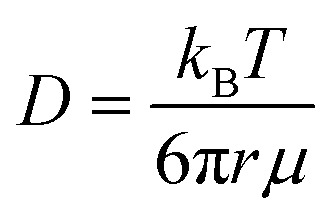
where *D* is the water self-diffusion coefficient [cm^2^ s^−1^], *k*_B_ is the Boltzmann constant (1.38 × 10^−23^ J K^−1^), *T* is absolute temperature (293 K), *r* is the effective radius of a diffusing water molecule [Å], and *μ* is the dynamic viscosity of the solution [Pa s]. For pure water, the Stokes–Einstein relation yields a water radius of 0.97 Å based on the measured self-diffusion coefficient of pure water (2.20 × 10^−5^ cm^2^ s^−1^) and the dynamic viscosity of pure water (1.01 × 10^−3^ Pa s); we assume this radius is constant at all solution concentrations. Dynamic viscosities of aqueous PEO solutions at 20 °C are taken from the literature.^51^

The water diffusivities derived from the Stokes–Einstein relation decrease exponentially with increasing PEO concentration by two orders of magnitude from 2.2 × 10^−5^ cm^2^ s^−1^ for pure water to 1.5 × 10^−7^ cm^2^ s^−1^ for 90 wt% PEO owing to the increase in solution viscosity (*cf.*, Fig. 2B). However, the measured water diffusivities by PFG-NMR decrease only by a single order of magnitude over the same concentration range and exceed the Stokes–Einstein-derived diffusivities by a factor of 10 in the most concentrated solutions (90 wt% PEO). Even in dilute solutions below the PEO overlap concentration, time-averaged water diffusivities exceed those predicted by solution viscosity when the solution is treated as a hydrodynamic continuum.

The substantial deviations between the experimental data and the Stokes–Einstein model from dilute to concentrated solutions suggest that a molecular rather than continuum view of polymer solutions is necessary for capturing the relevant mechanics of the dynamics of water interacting with the polymers. The Stokes–Einstein relation treats polymer solutions as a homogeneous medium comprised of spherical, non-interacting particles, an assumption that severely underpredicts the actual time-averaged water diffusivities. In solutions with a high concentration of PEO, the increase in internal friction between solution components increases the fluid viscosity by around two orders of magnitude, due in part to increased polymer–polymer interactions. We hypothesize that the self-diffusion behavior of the smaller water molecules is somewhat decoupled from and less affected by the slower coordinated motion of polymer chains, and thus exhibits a weaker concentration dependence. This hypothesis is consistent with previous studies that found faster penetrant diffusion than predicted by Stokes–Einstein in liquids when the diffusing species is much smaller than the molecules that comprise the bulk fluid phase and is of a similar size as the void structure in the dense liquid.^52,53^ These observations suggest the need to consider diffusion models that explicitly account for such molecular structure, or free volume, in polymer solutions.

#### Free volume models

We find that a model based on Fujita's free volume theory does, in fact, contain sufficient molecular detail to capture time-averaged water self-diffusion coefficients in both dilute and concentrated PEO solutions (*cf.*, Fig. 2A). It was postulated over a century ago that sufficient free volume between molecules must exist for diffusion or flow to occur in liquids, and that the viscosity of liquids is inversely proportional to the amount of free volume within the liquid.^54^ Free volume theory has since been refined in the form of empirical relationships between dimensionless fractional free volume (FFV) and small molecule diffusivity in polymers and polymer solutions. FFV can be defined as the fraction of a solution's volume that is unoccupied by the molecular volumes of the solution components and which is available for small molecules, such as water, for diffusion *via* rearrangement of the solution components driven by thermal fluctuations.^38,55,56^ The free volume model we employ is^2^2
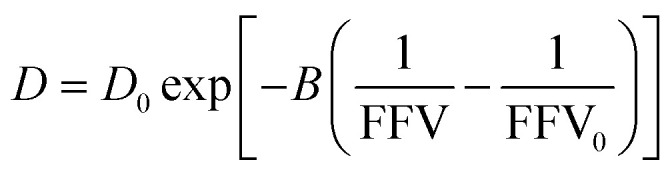
where *D*_0_ is the self-diffusion coefficient of pure water (2.20 × 10^−5^ cm^2^ s^−1^), *B* is an empirical fitting parameter, FFV is the fractional free volume of the PEO solution at a given concentration estimated from solution density, and FFV_0_ is the fractional free volume of pure water, here assumed to be 0.251 based on a water van Der Waals volume of 0.577 cm^3^ g^−1^.^57^*B* is typically proportional to the radius squared of the diffusing species and is here regressed to a value of 0.82 using PFG-NMR diffusivities.^58^ This model can be derived by assuming small molecule diffusivity is proportional to the probability of finding a free volume cavity larger than the diffusing species in a mixture containing a distribution of free volume element sizes. Detailed derivations for this expression and solution FFV values are provided in the ESI.† Because FFV is derived from solution densities and van Der Waals volumes of solution components, FFV implicitly accounts for molecular interactions insofar as they affect the density and packing of molecules in solution.

As shown in Fig. 2B, we find this free volume model well-describes water diffusion across the entire concentration range (0 to 90 wt% PEO). FFV values estimated from solution densities decrease with increasing PEO concentration from 0.251 for pure water to 0.129 for pure PEO due in part to an excess volume of mixing (Fig. S2A†) and in part to the lower FFV of pure PEO compared to that of pure water. These results suggest that slower water diffusion in concentrated PEO solutions primarily arises from a reduction in free volume between water molecules and PEO chains. This behavior is further consistent with previous observations of water diffusion in polymer networks.^2,59^ Free volume theory accounts for molecular packing of species in solution and appears to capture sufficient molecular detail to model water diffusion, not only in dilute PEO solutions but also above the polymer overlap concentration into highly concentrated solutions that resemble dense polymer networks. It should also be acknowledged that free volume itself is a nuanced concept, treated by some authors as an entropic quantity representing the probability of creating a diffusion channel through a discontinuous and fluctuating distribution of voids in a liquid,^38,60^ and is also considered to have both a static and dynamic component.^60–62^ Yet despite the subtle complexities of free volume theory, the simple empirical expression in eqn (2) is still remarkably effective at capturing water diffusion behavior across a wide range of PEO solution concentrations.

**Fig. 3:**
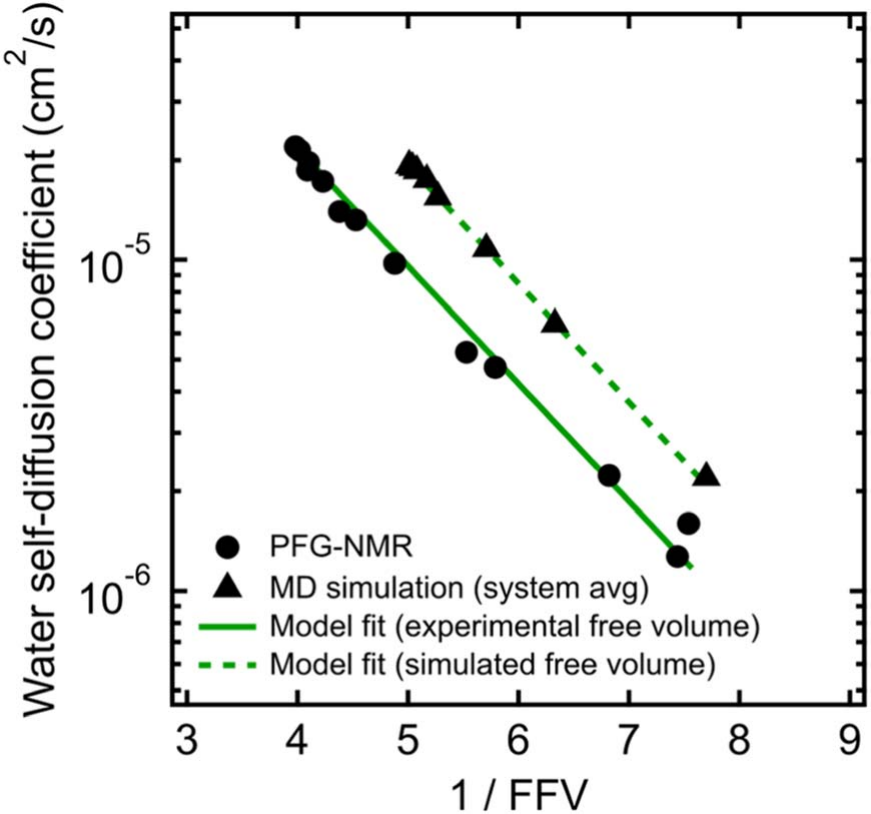
Free volume model for water self-diffusion coefficients in PEO determined by PFG-NMR using fractional free volume (FFV) values derived from experimental solution densities (black circles and solid green line) compared to free volume model fit for system-average water self-diffusion coefficients determined by MD simulations using FFV values derived from MD simulations (black triangles and dashed green line).

To validate that the improvement in this model is not simply due to the fact that it contains more fitting parameters than Stokes–Einstein, we directly compute FFV values in PEO solutions from MD simulations using an adapted procedure from Califano and coworkers.^63^ Rather than approximating FFV from macroscopic thermodynamic quantities (*i.e.*, solution densities) as performed above, these simulated FFV values are directly derived from atomistic configurational snapshots of PEO–water solutions. In brief, we insert a spherical test probe of radius 0.53 Å at the nodes of a grid and check for overlaps between the test particle and the atomic coordinates of a given configuration (further details are provided in the ESI†). Simulated FFV values agree qualitatively with experimentally-derived FFVs, while exhibiting ∼20% smaller values compared to those derived from solution densities (*cf.*, Fig. S1†). As discussed further in the ESI,† the magnitude of simulation-predicted FFVs depend on assumed values for the probe size; however, the relationship between the FFV and water self-diffusivity remains largely unchanged regardless of the probe size used. Furthermore, identical scaling is found between independently determined experimental and simulated sets of water diffusivities and FFV values as shown in Fig. 3 (*B* = 0.82 for PFG-NMR data and *B* = 0.83 for MD data). These findings reinforce the idea that the nanoscale structural properties of water in PEO solution are implicitly accounted for in the description of the free volume parameter, and that the varying FFV with increasing PEO concentration governs the water dynamics in PEO–water solutions at timescales of both picoseconds and milliseconds.

A simplification of the free volume model shown in eqn (2) was developed by Yasuda, who approximated FFV in hydrated polymer membranes as equal to the water volume fraction, *ϕ*_w_, of the swollen polymer.^9^3
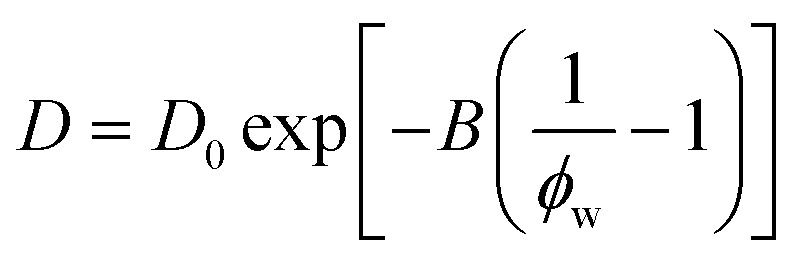


Eqn (3) implicitly assumes that water molecules are sufficiently mobile that they can exchange positions freely with each other in a highly swollen polymer membrane and that the volume occupied by water molecules is effectively “free” for water or solute diffusion at the macroscopic scale, whereas water molecular volumes are excluded from FFV in eqn (2). This expression also assumes, albeit unrealistically, that small molecule diffusion through a pure polymer cannot occur due to insufficient free volume. Thus, FFV is equated with *ϕ*_w_ in eqn (3) and is assumed to be zero for a pure polymer and unity for pure liquid water. As shown in Fig. 2B, Yasuda's approximation appears valid for dilute solutions at concentrations up to ∼45 wt% PEO where larger, more continuous pathways for water molecules exist yet significantly underpredicts water diffusivities in more concentrated solutions where polymer chains overlap. Yasuda's assumption of zero diffusivity in a pure polymer (*i.e.*, 100% PEO) due to insufficient free volume is not physically reasonable as PEO chains in reality are dynamic and imperfectly packed and not immovable, tightly packed obstacles, permitting water to move with a finite diffusivity even in highly concentrated solutions at polymer concentrations approaching that of the pure polymer liquid. Assigning non-zero values of FFV to the pure polymer in eqn (2) appears sufficient to account for such effects.

#### Mackie–Meares obstruction model

To compare findings from free volume theory with a simpler diffusion model that also accounts for the polymer structure, we consider the obstruction model developed by Mackie and Meares, which treats polymer chains as static obstacles to diffusion, which has successfully been applied to describe water and solute diffusion in highly swollen polymer networks.^10,64^ If the mobility of the polymer chains in solution is assumed to be much lower than that of water, as is the case in swollen networks, polymer chains could be seen as effectively increasing the tortuosity of the diffusion pathway of individual water molecules. The Mackie–Meares model thus relates the observed self-diffusivity to the self-diffusivity of pure water and a concentration-dependent tortuosity factor that varies from 0 to 1:4
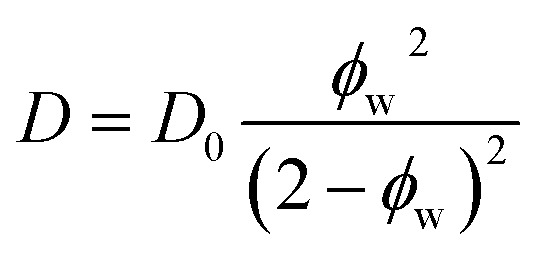
where *D*_0_ is the self-diffusion coefficient of pure water (2.20 × 10^−5^ cm^2^ s^−1^), and *ϕ*_w_ is the volume fraction of water in solution.

In dilute solutions up to ∼20 wt% PEO, the PFG-NMR-derived water self-diffusivities closely follow the predictions of the Mackie–Meares model (*cf.*, Fig. 2B). However, above 20 wt% PEO, water self-diffusion is faster than the model predicts, especially for highly concentrated solutions where the data and model deviate widely. Like Yasuda's approximation to the free volume model, Mackie–Meares predicts an unrealistic zero water diffusivity at zero water volume fraction (*i.e.*, pure PEO). The fact that the Mackie–Meares model also underpredicts water diffusivities at intermediate concentrations (20 to ∼60 wt% PEO) indicates that the tortuosity factor does not sufficiently describe the effect of solution structure on dynamics in more polymer-rich mixtures and that more explicit structural information, such as free volume, is necessary to describe water self-diffusion in semi-dilute and concentrated PEO solutions.

### Water diffusion in aqueous glycerol solutions

**Fig. 4:**
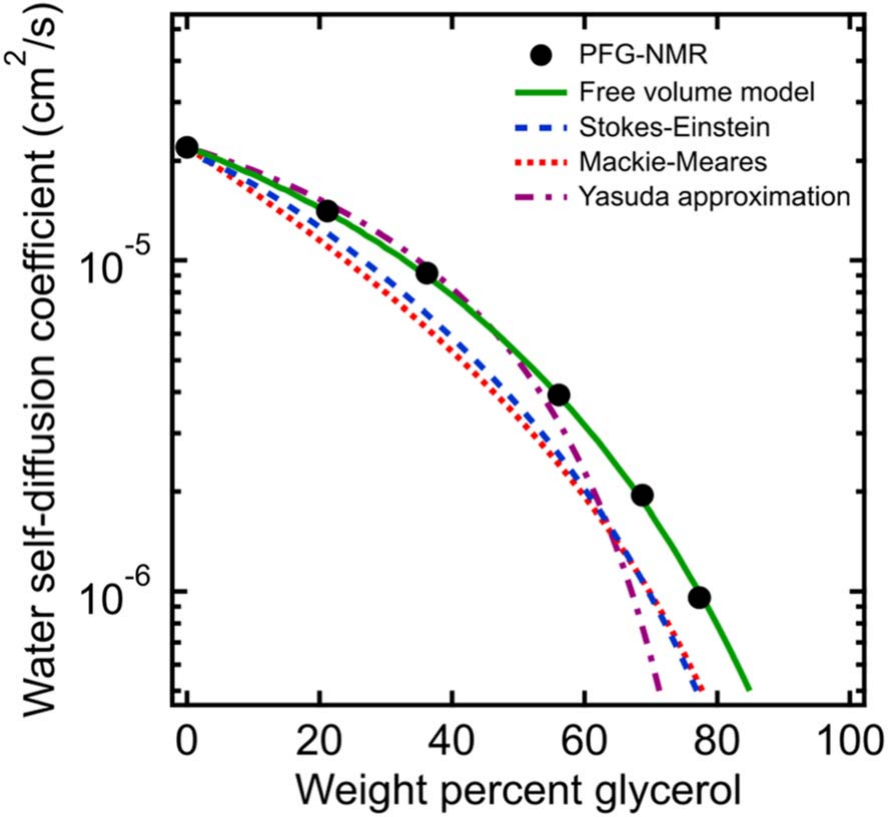
Comparison of PFG-NMR water diffusivities in aqueous glycerol solutions (black circles) with model fits for Stokes–Einstein (blue dashed curve), Mackie–Meares (red dotted curve), free volume theory (green solid curve), and Yasuda's free volume approximation (purple dashed/dotted curve).

To compare the behavior of PEO oligomer solutions with that of even simpler small molecule solutions, we also measured time-averaged water self-diffusivities in aqueous glycerol solutions using PFG-NMR across a similar concentration range of 0 to 77 wt% glycerol. Fig. 4 shows water self-diffusivities in glycerol solutions decrease exponentially with concentration from the value of pure water (2.2 × 10^−5^ cm^2^ s^−1^) to 9.6 × 10^−7^ cm^2^ s^−1^ at 77 wt% glycerol. Water diffusivities in glycerol solutions likewise diverged from the Stokes–Einstein and Mackie–Meares models at high glycerol concentrations, but were well-fit by the free volume model in eqn (2) (*cf.*, Fig. 4). These findings further indicate that molecular structure affects diffusion not only in polymer solutions but even in aqueous solution of small molecules.

### Nanoscale water dynamics from ODNP experiments

We next investigate water's nanoscale local diffusion in PEO solution using Overhauser Dynamic Nuclear Polarization (ODNP). ODNP experiments selectively capture translational water dynamics at picosecond to nanosecond timescales (*cf.*, Fig. 1), in contrast to PFG-NMR, which captures dynamics ∼10^9^ times faster and averaged over the entire system. ODNP isolates local water dynamics within approximately 1 nm of a spin label tethered to the PEO chain end, thus achieving surface selectivity, and has previously been demonstrated near molecular or extended surfaces of proteins, lipid membranes, and silica nanoparticles.^41,65,66^ ODNP is a dual NMR/EPR technique performed using paramagnetic nitroxide-based spin labels tethered to molecules and surfaces that are immersed in water. Saturating the EPR transition of the electron spins of the spin labels results in hyperpolarization of the ^1^H nuclear spins of waters that are within a ∼1 nm with an efficiency that depends on the lifetimes of the dipolar interactions between the electrons and ^1^H spins. Thus, the diffusion of waters (roughly on the order of the ^1^H nuclear spin *T*_1_ relaxation time) sets the efficiency of the hyperpolarization and correspondingly enhances the ^1^H NMR signal. Analyzing the trend in ^1^H NMR signal as a function of the extent of saturation of the EPR transition allows one to extract diffusion coefficients by fitting the trend to analytical expressions. Details of ODNP theory, instrumentation, experimental procedures, and data analysis strategies are discussed at length in previous studies,^67–71^ and details specific to this work are provided in the ESI.† In the present work, ODNP provides an essential contrast to PFG-NMR due to its nanoscale spatial resolution to capture water diffusion exclusively within the hydration shell of PEO, and this work further represents the first attempt to use ODNP in molecularly-crowded, concentrated polymer solutions.

**Fig. 5:**
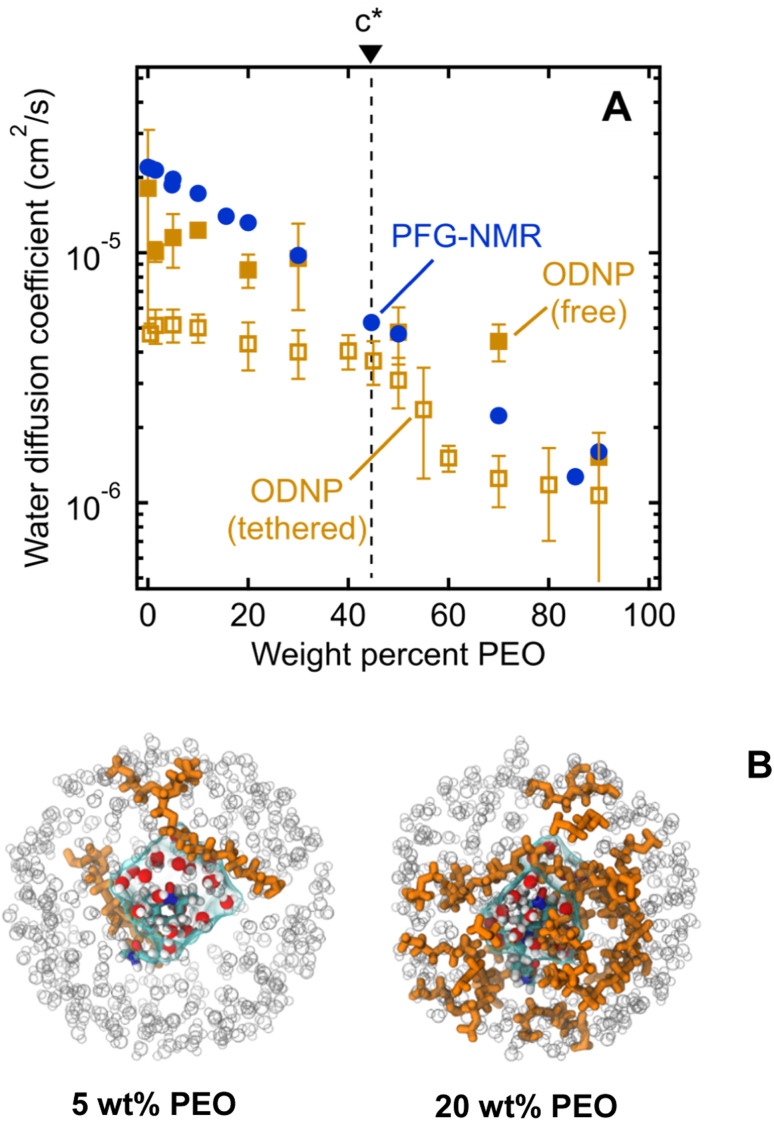
(A) Comparison of water diffusion coefficients in PEO solutions determined by PFG-NMR (filled blue circles), ODNP with PEO-tethered spin labels (unfilled gold squares), and ODNP with free TEMPOL spin labels (filled gold squares). PEO overlap concentration (c*) is marked by a dashed black line. (B) MD snapshots illustrating first hydration waters around TEMPO spin label on PEO chain end for 5 and 20 wt% aqueous PEO solutions. Orange molecules represent PEO chains, grey circles represent water molecules beyond first hydration layer, and cyan surface represents volume enclosing first hydration shell waters.

We observe ODNP-derived water diffusion coefficients, *D*_ODNP_, that scale with polymer concentration from 4.7 × 10^−6^ cm^2^ s^−1^ for 0.5 wt% PEO to 1.1 × 10^−6^ cm^2^ s^−1^ for 90 wt% PEO solutions (Fig. 5A). All PEO solutions used for ODNP measurements contain 200 μM spin-labeled PEO, supplemented with unlabeled PEO (MW = 550 g mol^−1^) and were measured at 18 °C. We find that the trend in *D*_ODNP_ is essentially invariant with PEO concentration in the dilute polymer regime, in contrast with the PFG-NMR and MD-derived water diffusivities. Only above 45 wt% PEO, *D*_ODNP_ begins to decrease with polymer concentration, in parallel with PFG-NMR-derived diffusivities. Across all concentrations, ODNP reports on slower water diffusivities compared to PFG-NMR and MD simulation.

We hypothesize that ODNP detects slower water diffusion due to its isolation of water dynamics within the hydration shell of PEO. Qualitatively, this agrees with the observation from MD simulations which show a 20–30% slower diffusion coefficient when isolating waters within 8 Å of the PEO chain end, as illustrated in Fig. 5B. Because PFG-NMR measures water diffusion over ∼10^9^ longer timescales than ODNP, diffusing water molecules are able to sample a large number of solvation sites in solution, including those not directly bound to PEO. We note that the crossover in *D*_ODNP_ trends between the dilute polymer regime to the concentrated polymer regime, where *D*_ODNP_ begins to decrease, occurs at the polymer overlap concentration, c*.^24,44,45^ This suggests that in crowded polymer solutions above c*, bulk and surface dynamics of water become indistinguishable. Above c*, hydration shells around neighboring PEO chains are close enough to share water molecules. The discontinuity in *D*_ODNP_ at c* may be the result of a molecular crowding effect that is felt by individual water molecules local to the polymer chains that is averaged out in the PFG-NMR-derived diffusivities where no significant transition at c* is observed.

We also perform ODNP experiments using a small molecule 4-hydroxy-2,2,6,6-tetramethylpiperidine 1-oxyl (TEMPOL) spin label dissolved into the bulk solution of PEO and water (*i.e.*, the spin label is not tethered to the PEO chain end) for the purpose of observing ODNP-derived water diffusion from the entire solution, not exclusively local to the PEO chains. We observe systematically higher water diffusivities with free TEMPOL than PEO-labeled systems, in semi-quantitative agreement with the system-averaged PFG-NMR data (Fig. 5A). This finding further supports the interpretation of PEO-labeled ODNP as capturing the slowed water dynamics of the hydration shell near the polymer surface. We note that the spin label has a minimal effect on the ODNP water dynamics and is not responsible for the observed trends (see ESI† for more details). Furthermore, the ODNP-derived diffusivity on PEO-labeled surfaces is independent of the MW of PEO (see ESI†). This is consistent with the concept that the deviation of water diffusivity from simple fluid dynamic models originates from PEO–water interactions at the molecular level that are not altered by the molecular weight of PEO.

While the slowing of water dynamics near PEO represents the most likely explanation of ODNP results, additional nuances of the ODNP experiment must also be considered when interpreting these data. Experimentally derived ODNP coupling factors depend on the choice of appropriate electron spin saturation factors (*s*_max_), which affect the absolute values of *D*_ODNP_ but are technically challenging to obtain. Values of *s*_max_ range between 1/3 to 1 depending on Heisenberg exchange rates between colliding nitroxide groups and ^14^N nuclear spin relaxation rates.^70,72^ Typically for systems with the spin label tethered to a macromolecule, a value of 1 for *s*_max_ is appropriate due to fast ^14^N relaxation that leads to saturation over all three ^14^N nitrogen hyperfine-split manifold of the EPR signal in ODNP experiments. Hence, *s*_max_ = 1 was used for all spin-labeled PEO measurements. Saturation factors are approximated as independent of polymer concentration in the present study. Determining concentration-dependent saturation factors in experiments utilizing free TEMPOL in future studies could slightly improve the accuracy of *D*_ODNP_, potentially changing diffusion values by as much as 10% if *s*_max_ values were found to be significantly different (see ESI† for estimation of *s*_max_ sensitivity).

Water and PEO diffusivities determined by PFG-NMR are specifically self- (or intra-) diffusion coefficients, which represent the rate at which identical molecules interchange at equilibrium in the absence of substantial concentration gradients.^73^ Mutual (or inter-) diffusion coefficients, in contrast, represent the rate at which random thermal motion of molecules equilibrate concentration gradients or fluctuations, and have been measured for aqueous PEO solutions *via* techniques such as Taylor dispersion.^73^ ODNP-derived water diffusivities specifically represent the “relative instantaneous” diffusion rates of water and spin-labeled molecules (free TEMPOL or PEO-tethered TEMPO) that are both undergoing independent Brownian motion.^70,74–76^ To compare with ODNP water diffusivities, concentration-dependent water–PEO mutual diffusion coefficients can be estimated from PFG-NMR-derived water and PEO self-diffusion coefficients as well as literature data on water activity coefficients for aqueous PEO solutions (see ESI†).^77^ Close agreement is found between water–PEO mutual diffusion coefficients and ODNP-derived water–PEO relative instantaneous diffusion coefficients in both dilute and concentrated solutions, which may indicate similarities between relative translational motion of water and spin-labels at both molecular and macroscopic scales. However, this agreement may be serendipitous. ODNP diffusivities and mutual diffusivities represent different quantities, since ODNP specifically captures water dynamics near a spin-labeled surface, while mutual diffusivities represent the rates at which concentration gradients or fluctuations relax toward equilibrium in the bulk. In highly dilute solutions, the mutual diffusion coefficient between water and large macromolecules such as DNA or proteins approaches the self-diffusion coefficient of the macromolecule (∼10^−7^ to 10^−6^ cm^2^ s^−1^ for DNA),^78–80^ which is substantially slower than ODNP water diffusivities that have been measured near macromolecule surfaces (∼10^−5^ cm^2^ s^−1^ for DNA).^40,41,81^

### Structural ordering of water revealed by MD simulations

**Fig. 6:**
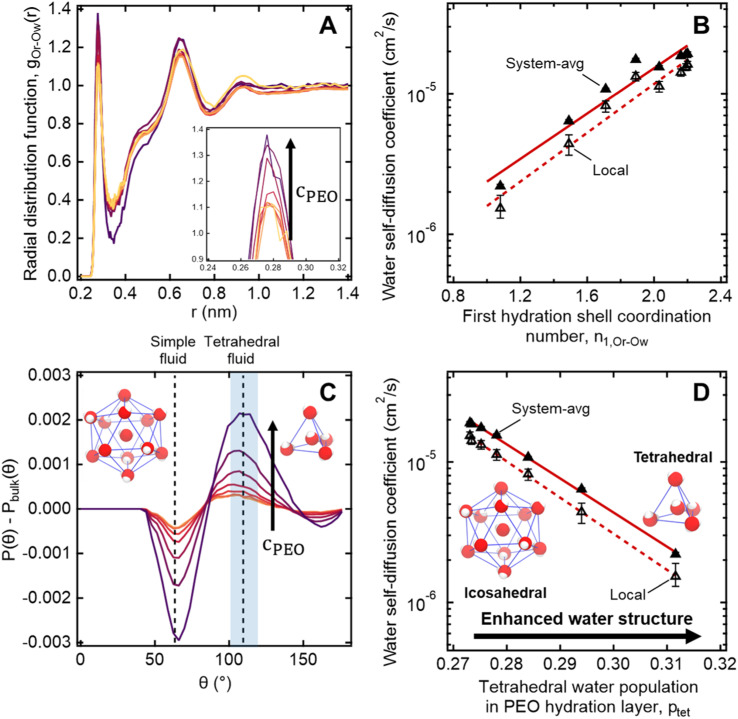
(A) Radial distribution functions of water molecules near PEO chains maintain similar shapes with increasing PEO concentration from 0.5 to 50 wt% with a systematic increase in the 1st and 2nd peak heights (darker colors correspond to higher concentrations). (B) MD-derived local and system-averaged water self-diffusivities in PEO solutions generally correlate with the coordination number of the first hydration shell. (C) Three-body angle distributions show enhancement in the tetrahedrality of water (109.5°) in the hydration layer near PEO chains with increasing PEO concentration from 0.5 to 50 wt% (darker colors correspond to higher concentrations). (D) The integral over the tetrahedral region of the three-body angle distributions *P*(*θ*), indicated by the shaded region in part A, correlates with both local and system-averaged water self-diffusivities in PEO solutions derived from MD simulations.

To further investigate the connection between molecular structure and water dynamics, we employ MD simulations to probe the effect of PEO crowding on the structure of hydration waters. First, we compute 2-D radial distribution functions (RDFs) to determine how PEO concentration affects local water density near PEO chains relative to that of the bulk solution. Specifically, we consider the RDFs *g*(*r*_Or−Ow_) between the radical oxygen on the spin probe (*O*_r_) and all nearby water oxygens (*O*_w_), where *r*_Or−Ow_ is the distance between a radical oxygen and water oxygen. Shifts in RDF peak position and amplitude with concentration indicate changes in the local environment such as changes in temperature, concentration, and water affinity (*i.e.*, hydrophobicity). For the entire concentration range of 0 to 50 wt% PEO, RDFs do not exhibit changes in overall peak shape or location (*cf.*, Fig. 6A). Rather, there is a systematic increase in the amplitude of the first peak around 0.28 nm with increased PEO concentration, indicating an enhancement in the number density of water near the spin probe relative to bulk solution, where we define the bulk region as *r*_Or−Ow_ > 1.5 nm. We hypothesize that this increased local number density of water relative to bulk solution decreases the local free volume, effectively slowing water diffusion in the hydration layer below the bulk value.

We further quantify local water density correlations by computing the coordination number of the first hydration shell, *n*_1,Or−Ow_. Values for *n*_1,Or−Ow_ are estimated by integrating *ρg*(*r*_Or−Ow_) over *r*_Or−Ow_ from 0 to 0.34 nm (*i.e.*, the position of the first RDF minimum), where *ρ* is the number density of water in the bulk. *n*_1,Or−Ow_ decreases from 2.2 to 1.08 as PEO concentration increases from 0 to 50 wt% as neighboring PEO chains begin to crowd out water molecules in the first hydration shell. As shown in Fig. 6B, we observe a general correlation between both local and system-average water self-diffusivities and *n*_1,Or−Ow_ (*R*^2^ = 0.94), suggesting changes in water structure near PEO chains affect local water dynamics that in turn affect dynamics averaged over the bulk. While metrics like the FFV and *n*_1,Or−Ow_ hint at systematic shifts in water structure, these metrics do not directly probe water's tetrahedral network structure.

To more directly describe changes in the tetrahedral structure of PEO–water, we compute three-body angle distributions *P*(*θ*), which present a detailed picture of water coordination by capturing the effects of small shifts in the solution environment on water structure.^82–84^ To construct 3-body angle distributions, we compute and histogram the angles between hydration water oxygens (*i.e.*, those within 4.2 Å of PEO heavy atoms) and their two nearest neighboring water oxygens. Peaks in Fig. 6C represent differences between water populations of a particular coordination local to PEO chains in solution from those in pure water. We observe peaks at 64° and 109.5°, corresponding to a decrease in the population of icosahedrally-coordinated hydration waters near the PEO chains and an increase in the population of tetrahedrally-coordinated hydration waters, respectively. A shift from icosahedral to tetrahedral coordination indicates a shift away from simple fluid behavior toward orientations typically associated with enhancements in water ordering under supercooling^85^ and in the hydration layers of small hydrophobic molecules.^86^ As PEO concentration increases, the hydration layer exhibits a monotonic increase in the population of tetrahedrally-coordinated waters and a monotonic decrease in the population of icosahedrally-coordinated waters (*cf.*, Fig. 6C).

To directly quantify the increase in hydration water ordering with PEO concentration, we approximate the population of tetrahedrally-coordinated waters as 
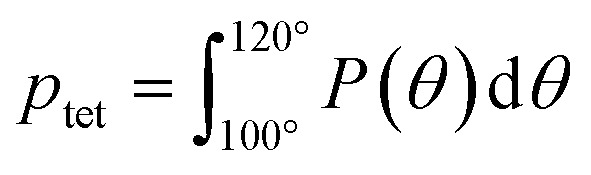
. For the entire range of PEO concentrations, there is a substantial increase in tetrahedral ordering as *p*_tet_ increases by 5% from 0 to 50 wt% PEO solutions, indicating an enhancement in the water network structure near PEO chains (*cf.*, Fig. 6D). Both local and system-averaged water self-diffusivities in PEO solutions from MD correlate inversely with the population of tetrahedral ordered hydration waters (*cf.*, Fig. 6D; *R*^2^ > 0.99). The relationship between equilibrium water dynamics and structure in PEO–water has been previously discussed in the computational literature.^16,17^ For instance, Borodin *et al.* demonstrated that water self-diffusivity in PEO–water can be reconstructed from the portion of waters hydrogen-bonded to a PEO ether oxygen. While water–water hydrogen bonding is closely related to water's tetrahedral structure, metrics of tetrahedrality do not directly report on hydrogen bonding. We find that *p*_tet_ increases and the average number of water–water hydrogen bonds per water molecule decreases with increasing PEO concentration (see ESI†). This suggests that even as water molecules increasingly favor hydrogen bonding with PEO, the tetrahedral network structure of water persists.

The structure-dynamics connection apparent in Fig. 6D paired with the literature, suggests that, in addition to reduced free volume and changes in local water density, enhanced tetrahedrality in more concentrated solutions leads to the concentration-dependent retardation of hydration water translational dynamics. Remarkably, water's network structure appears enhanced local to hydrophilic PEO, a phenomenon usually attributed to small hydrophobic molecules and moieties.^86,87^ This is in keeping with recent computational studies that demonstrated a more general enhancement water tetrahedrality in the hydration layer of various small length-scale (<1 nm) chemical moieties irrespective of the chemical identity.^84,88^

## Conclusions

A unique set of complementary experimental and simulation tools reveals the important role of molecular surfaces and structure on water dynamics in polymer solutions. Local and bulk water dynamics both decrease with increasing PEO concentration and converge above the polymer overlap concentration due to molecular crowding effects, revealed by Pulsed-Field Gradient (PFG) NMR and Overhauser Dynamic Nuclear Polarization (ODNP) experiments that probe water dynamics at macroscopic and molecular scales, respectively. Polymer concentration affects water diffusion at both scales by changing water structural properties that alter free volume. Specifically, enhanced tetrahedral ordering and densification of water near oligomeric PEO chains observed by Molecular Dynamics (MD) simulations in aqueous solutions leads to slowed local water diffusion near polymer chains relative to diffusion in the bulk. Our study shows that free volume theory captures time-averaged water diffusion behavior in solution, which is not fully described by solely continuum models such as Stokes–Einstein, since free volume theory implicitly accounts for molecular structural features in polymer solutions. We highlight the success of free volume theory at capturing diffusion in the concentrated polymer regime, which most closely represents the polymer and water contents realized in conventional dense polymer membranes. Understanding the structural and dynamic behavior of water in polymer solutions lays the groundwork for developing molecularly-informed design rules for more complex polymer materials such as membranes used for separation and filtration. The ability to tune or program water transport through rational design of surface chemistry and topology could enable design of advanced membranes to treat recalcitrant water resources.

## Materials and methods

### Pulsed-field gradient (PFG) NMR

All ^1^H pulsed-field gradient (PFG) NMR experiments were performed on a 300 MHz (7.05 T) SWB Bruker NMR spectrometer with a Diff50 probe, fitted with a 10 mm ^1^H coil. Aqueous PEO solutions in deionized water were prepared at desired mass ratios from poly(ethylene glycol) methyl ether (Aldrich, 550 g mol^−1^), which were then transferred into standard 5 mm NMR tubes and sealed with parafilm. Precise temperature control was achieved using the standard Bruker gas temperature control system, with a flow of N_2_ gas at a rate of 800 L h^−1^. The temperature was maintained at 21 °C calibrated using dry methanol and dry ethylene glycol at sub-ambient and elevated temperatures, respectively. The power level used for the ^1^H on the Diff50 probe was 40 W with a 90° pulse duration of around 18.5 μs (13.5 kHz). For all measurements, a recycle delay of 5*T*_1_ was applied before each scan, to allow full relaxation. The ^1^H chemical shift was calibrated using a dry methanol sample using the CH_3_ peak (3.3 ppm).

The PFG-NMR experiments employed a diffusion sequence which includes a stimulated echo modification to protect the signal from *T*_2_ relaxation and bipolar pulses to avoid any effects from imperfections. The diffusion was measured using a variable magnetic field gradient strength sequence, where the maximum gradient available for this hardware setup was 2800 G cm^−1^. The selection of gradient strength was chosen for each measurement to ensure an appropriate window on the decay curve was acquired. For all measurements the value of the gradient duration (*δ*) and diffusion time (*Δ*) were set to 1 ms and 20 ms, respectively. Sample volumes were kept to a minimum to avoid any complications from temperature gradient induced convection.

### Overhauser dynamic nuclear polarization (ODNP) NMR relaxometry

Sample mixtures for ODNP were prepared by mixing water (Milli-Q purity) with 550 g mol^−1^ PEO at varying concentrations (0–90 wt%) including a constant 200 μM TEMPO end-labeled PEO (785 g mol^−1^). The TEMPO end-labeled PEO, or spin-labeled PEO, was prepared by reacting methoxy-terminated *m*-PEG13–NHS ester (BroadPharm BP-22584) with 4-amino-TEMPO (Sigma-Aldrich 163945, used as purchased) in tetrahydrofuran with triethylamine as a catalyst, following the synthesis procedure reported previously in Sherck *et al.*^24^ Samples of 4 μL were loaded into round quartz capillaries of 0.6 mm ID × 0.84 mm OD (Vitrocom, New Jersey, USA), sealed on one end with Critoseal and on the other end with melted beeswax. ODNP experiments were performed on a Bruker EMXPlus spectrometer with a Bruker Avance III NMR console (Bruker, Massachusetts, USA). The sample capillaries were mounted on a home-built NMR probe with a copper U-shaped rf coil centered in a high sensitivity microwave cavity (Bruker ER 4119HS-LC). Experiments were performed at 9.8 GHz (X-band) microwave frequency and a center magnetic field of 3484 G, coinciding with the center resonance of the nitroxide EPR spectrum. Dry air (20 SCFH) was streamed through the cavity across the probe and capillary to maintain a temperature of 18 °C. Theory outlining the experiment and data analysis including calculation of the cross-relaxation rate, *k*_σ_, coupling factor, *ξ*, and diffusion coefficient are detailed in previous work.^40,41,69,70^

### Computational methods

We applied molecular dynamics models composed of the OPC 4-site water model,^89^ a generalized AMBER forcefield (GAFF2)^90,91^ parametrized model for PEO,^24^ and a TEMPO spin probe functionalized PEO molecule. Both the OPC water model and the PEO model accurately reproduce the thermophysical properties of pure water and PEO under ambient conditions (298.15 K and 1 bar). Previous work demonstrated the ability of this functionalized PEO model to accurately describe the conformational landscape of PEO in close agreement with findings from experimental Double Electron-Electron Resonance (DEER) spectroscopy.^24,92^ The partial charges of the spin probe-functionalized PEO were obtained using the AMBER18 Antechamber package^93^ informed by quantum chemical calculations using the Gaussian 16 software^94^ as described in our previous work.^24^ All other inter- and intramolecular parameters came from the second-generation generalized AMBER forcefield (GAFF2).^90,91^ The results of this parametrization scheme yielded similar parameters to those obtained in previous work.^95^ All coulombic interactions were described with particle-mesh Ewald summation scheme (PME).^96^

We considered PEO–water compositions of 0, 0.5, 1.5, 5, 10, 20, 33 and 50 wt% PEO using the GPU-optimized OpenMM molecular simulation software.^97^ Each system was first energy minimized to remove overlapping atom positions in our PACKMOL^98^ generated initial configurations. Systems were then equilibrated in the NPT ensemble using a Langevin Thermostat^99^ paired with a Monte Carlo barostat^99^ at 290 K and 1 bar. We determine the minimum necessary system equilibration period by estimating the time to convergence of the density and temperature (under 1 ns for all systems). Following equilibration, the NPT run continued for 200 ns with system configurations saved every 10 ps. NPT-generated trajectories were used to calculate structural properties of the PEO–water mixtures such as radial distribution functions, fractional free volumes (FFVs), and 3-body angle distributions. In addition to the system configurations, simulation states (atom positions and velocities) were saved every 10 ns. Each of these saved states served as the starting point for an independent 1 ns NVE simulation with system coordinates saved every 0.1 ps.

System-average water self-diffusivity, *D*_H_2_O_, values are estimated from the results of the 10 separate NVE simulations *via* the slope of the mean-square displacement (MSD) curve at long times
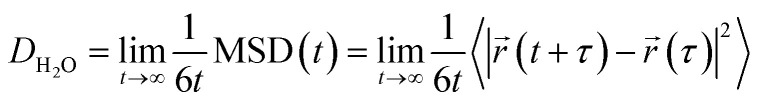
where *r⃑*, *t*, and *τ* are the position vector of a water oxygen, the current time step, and the initial time step. Here, 〈·〉 denotes the ensemble average of a quantity across all water positions. We carry out the MSD slope determination for PEO heavy atoms to estimate PEO self-diffusivity, *D*_PEO_. We compute the hydration layer water self-diffusivity, *D*_local_, by considering the MSD of waters residing within the first two hydration shells of the PEO spin probe (8 Å) rather than all system waters. We estimate the local MSD slope in the diffusive region *t* ∈ [10, 40] ps to mitigate the effect of the hydration waters leaving the vicinity of the spin probe. To estimate the uncertainty in water and PEO diffusivities, 95% confidence intervals were computed by bootstrapping the mean-squared displacement (MSD) curves obtained from 10 independent MD simulations.

## Data availability

All data are available upon reasonable request.

## Author contributions

Joshua D. Moon (conceptualization, data analysis and analytical modeling, writing – original draft); Thomas Webber (conceptualization, ODNP experiments and methodology, data analysis, writing – original draft); Dennis Robinson Brown (conceptualization, MD simulations and methodology, data analysis, writing – original draft); Peter M. Richardson (PFG-NMR experiments and methodology, data analysis); Thomas M. Casey (ODNP experiments and methodology, data analysis); Rachel A. Segalman (conceptualization, supervision, funding acquisition); M. Scott Shell (conceptualization, writing – review & editing, supervision, funding acquisition); Songi Han (conceptualization, writing – review & editing, supervision, funding acquisition).

## Conflicts of interest

There are no conflicts to declare.

## Supplementary Material

SC-015-D3SC05377F-s001
