# Supplementary material for: Nanoscale water–polymer interactions tune macroscopic diffusivity of water in aqueous poly(ethylene oxide) solutions
Source: Chem Sci. 2024 Jan 15;15(7):2495–508. doi: 10.1039/d3sc05377f (PMC10866362; doi:10.1039/d3sc05377f)
Supplement: SC-015-D3SC05377F-s001 [file SC-015-D3SC05377F-s001.pdf]

Electronic Supplementary Material (ESI) for Chemical Science.
This journal is © The Royal Society of Chemistry 2023

Supplementary Information for

Nanoscale water-polymer interactions tune macroscopic diffusivity of water in aqueous poly(ethylene oxide) solutions

Joshua D. Moon[†1,2], Thomas R. Webber[2], Dennis Robinson Brown[2], Peter M. Richardson[1], Thomas M. Casey[3], Rachel A. Segalman[1,2], M. Scott Shell[2], Songi Han[2,3]*

1. Materials Department, University of California, Santa Barbara, California 93106, United States
2. Department of Chemical Engineering, University of California, Santa Barbara, California 93106, United States
3. Department of Chemistry and Biochemistry, University of California, Santa Barbara, California 93106, United States

*Corresponding Author: Songi Han

Email: songi.han@northwestern.edu

[†]The present address of Dr. Joshua D. Moon is the Department of Chemical Engineering, University of Florida, Gainesville, Florida 32611, United States.

Supporting Information

Calculation of PEO's polymer overlap concentration....................2
Estimating fractional free volume for PEO and glycerol solutions 3
Fitting water self-diffusion coefficients to fluid mechanics and diffusion models 5
MD free volume and diffusion simulations....................8
ODNP measurement of water, PEO, and free TEMPOL....................10
Mutual diffusion coefficients....................11
MD simulations of solution structure....................14
Correlations between MD and ODNP water diffusivities and tetrahedral water population....................15
ODNP characterization of water dynamics in higher molecular weight PEO solutions 16
Tabulated water and PEO diffusion coefficients....................17
Supporting Information references 19

Calculation of PEO's polymer overlap concentration

There are several methods of obtaining the polymer overlap concentration. The most straightforward for a polymer in good solvent is to use the radius of gyration, R_g, as known from (1, 2):

$$c^* = \frac{MW/N_A}{\frac{4\pi}{3}R_g^3} \quad \text{Eqn. S1}$$

where the numerator is the mass of a single polymer chain in grams, and the denominator is the expected excluded volume occupied by the polymer chain in cubic nanometers.

From prior work by Sherck, et al.,(3) the scaling law for PEO end-to-end distance which spans the molecular weights used in this study was found to be:

$$R_{ee} = (0.047)MW^{0.588}\,nm \quad \text{Eqn. S2}$$

where the critical exponent 0.588 is that of a polymer in a good solvent, and the pre-exponential factor 0.047 is specific to PEO. The radius of gyration for polymers in a good solvent, such as PEO in water, is related to the end-to-end distance via:

$$R_g = \sqrt{R_{ee}^2/6.25} \quad \text{Eqn. S3}$$

For 550 g/mol PEO, the overlap concentration is 0.48 g/mL, or 46.4 wt%.

We note that alternate methods of obtaining the polymer overlap concentration, such as through application of the Flory-Fox equation with measured or estimated intrinsic viscosities (4–7) is an indirect method, only to be used when R_{ee} or R_g data is unavailable. Likewise, the use of dielectric spectroscopy measurements to obtain the overlap concentration is indirect, and not commonly reported in literature, owing to the complexity of application and inaccessibility to many research labs.

Estimating fractional free volume for PEO and glycerol solutions

Fractional free volume (FFV) can be defined for an aqueous PEO solution as the fraction of solution volume that is unoccupied by the molecular volumes of the solution components:

$$FFV = \frac{V_{free}}{V_{solution}} = 1 - \frac{V_{0,w} + V_{0,PEO}}{V_{solution}} \quad \text{Eqn. S4}$$

where V_{free} is the free volume of a solution, $V_{solution}$ is the total volume of the solution, and $V_{0,w}$ and $V_{0,PEO}$ are the occupied volumes of water and PEO, respectively. This expression can be written in terms of specific volumes:

$$FFV = 1 - \frac{w_w\hat{V}_{0,w} + w_{PEO}\hat{V}_{0,PEO}}{\hat{V}_{solution}} \quad \text{Eqn. S5}$$

where w_w and w_{PEO} are water and PEO weight fractions, respectively, $\hat{V}_{0,w}$ and $\hat{V}_{0,PEO}$ are specific volumes occupied by water molecules or PEO chains, respectively, and $\hat{V}_{solution}$ is the specific volume of the aqueous PEO solution (i.e., 1/density of the solution).(8–10) Specific occupied volume of a solution component, typically defined as the specific volume of the component at 0K, is commonly estimated for polymers as:

$$\hat{V}_{0,i} = 1.3\hat{V}_{vDw,i} \quad \text{Eqn. S6}$$

where $\hat{V}_{vDw,i}$ is the van der Waals volume of the polymer, and the 1.3 factor is an approximate "universal" packing factor relating the two volume terms.(11, 12) For polymers, $\hat{V}_{vDw,i}$ is often approximated using group contribution theory by summing the van der Waals volumes of individual polymer functional groups.(13–15) Using group contribution values given by Bondi, $\hat{V}_{vDw,PEO}$ for 550 g/mol PEO was estimated to be 0.594 cm^3/g, which yields a FFV for pure PEO of 0.129.(13) There is some degree of uncertainty in the literature about the most appropriate value to use for $\hat{V}_{vDw,w}$ for water.(8, 9, 16–20) The present study used a value of 0.577 cm^3/g based on the van der Waals volume of water in ice, which yields a FFV of pure water of 0.251.(18) Aqueous solution densities at 20 °C at varying concentrations of PEO with PEO molecular weights near 550 g/mol and at varying concentrations of glycerol were found in the literature.(21–23) Values of FFV for aqueous PEO solutions were calculated using Eqns. S5 and S6 and are shown in Figure S1. Non-linear trends of FFV with solution concentration are in part driven by a negative excess volume of mixing due to non-ideal mixing of water and PEO (cf., Figure S1).

FFV values for aqueous glycerol solutions were likewise determined, where $\hat{V}_{0,glycerol}$ is substituted for $\hat{V}_{0,PEO}$ in Eqns. S5 and S6. $\hat{V}_{0,glycerol}$ was determined to have a value of 0.724 cm^3/g using group contribution theory, yielding a FFV value for pure glycerol of 0.088. FFV values for glycerol solutions are also shown in Figure S1.

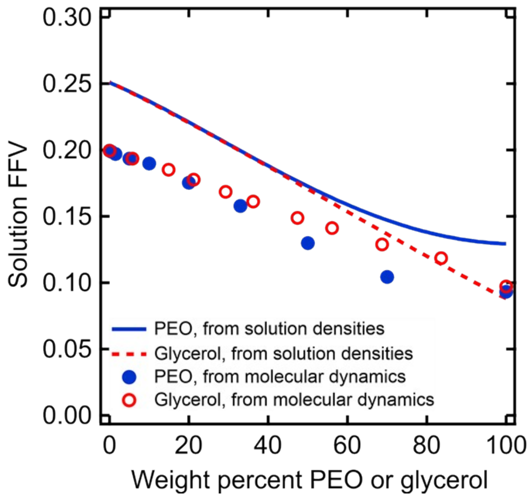

Figure S1. Comparison of fractional free volume (FFV) values derived from experimental solution densities (curves) and accessible FFV values simulated using Molecular Dynamics (circles) for PEO and glycerol solutions. Blue solid curve and filled circles are for PEO solutions, and red dotted curve and unfilled circles are for glycerol solutions.

Fitting water self-diffusion coefficients to fluid mechanics and diffusion models

Stokes-Einstein model

The Stokes-Einstein equation is given as:

$$D = \frac{k_B T}{6\pi r \mu} \quad \text{Eqn. S7}$$

where D is the water self-diffusion coefficient [cm^2/s], k_B is the Boltzmann constant (1.38 x 10^{-23} J/K), T is absolute temperature (293 K), r is the effective radius of a diffusing water molecule [Å], and μ is the dynamic viscosity of the solution [Pa·s]. The effective diffusion radius of water, r, was estimated to be 0.97 Å based on the measured self-diffusion coefficient of pure water (2.20 x 10^{-5} cm^2/s) and dynamic viscosity of pure water (1.01 x 10^{-3} Pa·s) and was assumed to be constant at all solution concentrations. Dynamic viscosities of aqueous PEO and glycerol solutions at 20 °C were found in the literature.(24, 25)

Mackie-Meares model

The Mackie-Meares equation is given as: (26)

$$D = \frac{D_0 \phi_w^2}{(2 - \phi_w)^2} \quad \text{Eqn. S8}$$

where D_0 is the self-diffusion coefficient of pure water (2.20 x 10^{-5} cm^2/s), and ϕ_w is the volume fraction of water in solution. Water volume fractions were determined as a function of solution composition using the partial specific volume of water and the mixture specific volumes of aqueous PEO or glycerol solutions:

$$\phi_w(w_w) = \frac{w_w \hat{V}_w(w_w)}{\hat{V}(w_w)} \quad \text{Eqn. S9}$$

where w_w is water weight fraction, $\hat{V}_w(w_w)$ is water partial specific volume [cm^3/g], and $\hat{V}$ is mixture specific volume [cm^3/g]. Water partial specific volumes were calculated at each value of w_w as the intercept of a vertical line at $w_w = 1$ to a tangent line drawn at w_w along a curve of $\hat{V}$ values plotted vs. w_w (cf., Figure S2A). Mixture specific volumes were given as the inverse of aqueous solution densities at 20 °C of 550 g/mol PEO or glycerol solutions at a given value of w_w.(21–23) Water volume fractions and FFV values determined using solution densities are shown in Figure S2B. Note the negative change in partial specific volume at higher concentrations in Figure S2A arising from the excess volume of mixing.

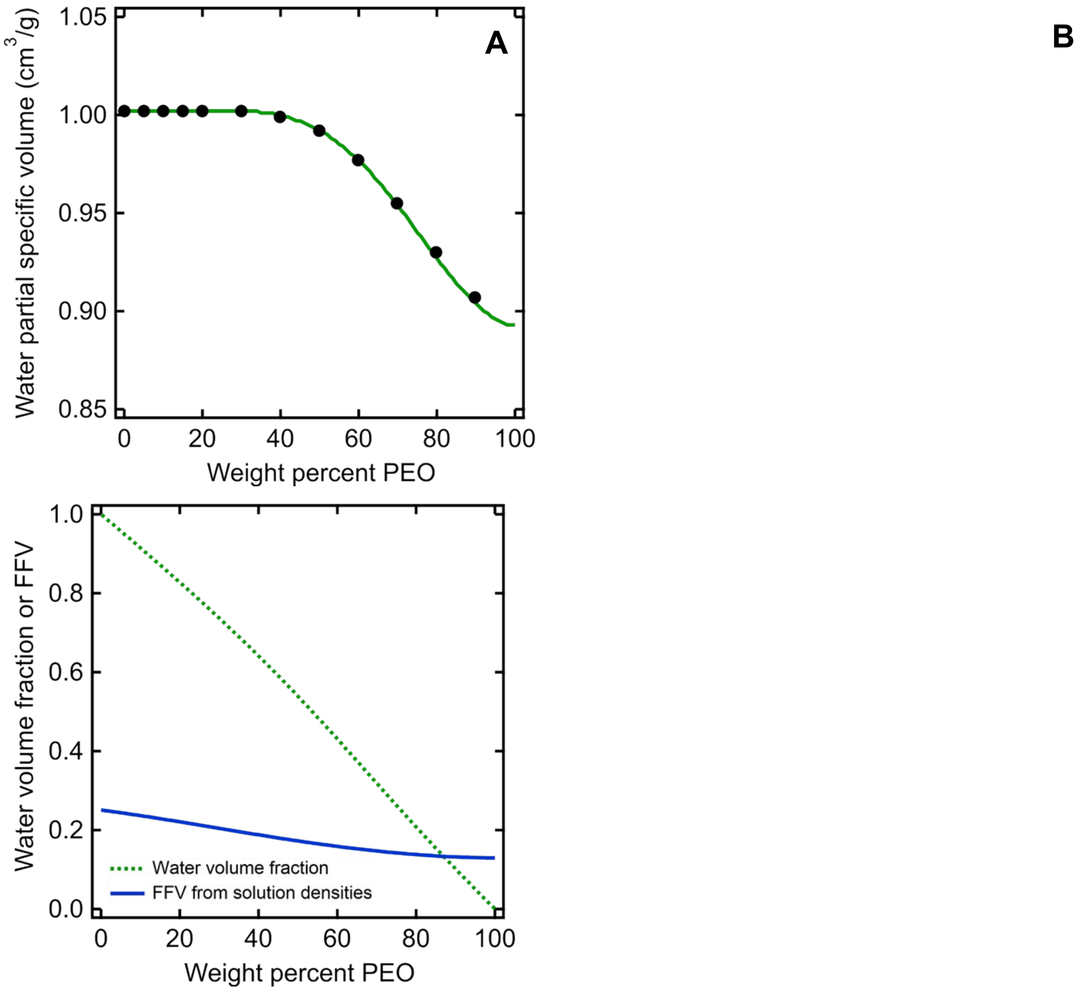

Figure S2. (A) Water partial specific volumes for PEO-water solutions as a function of PEO concentration derived from solution densities from Trivedi et al. using Eqn. S9.(21) (B) Comparison of water volume fractions (green dotted curve) determined using Eqn. S9 and FFV values (blue solid curve) determined from experimental solution densities for PEO solutions using Eqns. S5 and S6.

Free volume model

The empirical correlation between solvent self-diffusion coefficients and FFV that was largely developed by Vrentas and Duda is generally given as:(27–29)

$$D = A\exp\left(-\frac{B}{FFV}\right) \qquad \text{Eqn. S10}$$

where A [cm²/s] is a pre-exponential factor that is relatively insensitive to temperature, and B is a parameter related to the minimum local free volume required to be available for a diffusion jump to occur and is proportional to the size of the diffusing species. The pure solvent self-diffusion coefficient can be related to the FFV of the pure solvent as:

$$D_0 = A\exp\left(-\frac{B}{FFV_0}\right) \qquad \text{Eqn. S11}$$

For water, D_0 was measured by PFG-NMR to be 2.20 x 10^{-5} cm^2/s at 21 °C. By solving for A in Eqn. S11 and substituting the obtained expression into Eqn. S10, one can write an expression with a single fitting parameter to describe the interrelation between water self-diffusion in the solution and the fractional free volumes of the solution and pure solvent:(30)

$$D = D_0 \exp\left[- B\left(\frac{1}{FFV} - \frac{1}{FFV_0}\right)\right] \quad \text{Eqn. S12}$$

In Eqn. S12, the FFV term can be obtained from Eqn. S5 for each solution PEO concentration or weight fraction, and the FFV_0 term can be obtained from Eqn. S5 by setting water weight fraction to unity and was determined to have a value of 0.251 as discussed above.

The value of B was determined by the best fit of experimental PFG-NMR water self-diffusivities and corresponding solution FFV values to Eqn. S12 using non-linear least squares regression. For 550 g/mol aqueous PEO solutions at 21 °C, A and B were determined to have values of 5.73 x 10^{-4} cm^2/s and 0.82, respectively (R^2 = 0.994). For aqueous glycerol solutions at 21 °C, A and B were determined to have values of 4.60 x 10^{-4} cm^2/s and 0.76, respectively (R^2 > 0.999).

Yasuda approximation to free volume model

For the case of solute self-diffusion, such as sodium chloride, in highly swollen polymer membranes, Yasuda approximated FFV to be essentially equal to the volume fraction of water in the membrane based on the assumption of zero solute diffusivity in a pure polymer matrix.(31) This model also assumes ideal mixing of water and polymer. While this model was originally developed for salt or water soluble organic solute diffusion rather than solvent diffusion, one can nonetheless examine the applicability of this model for the case of water self-diffusion in PEO and glycerol solutions.(31, 32) This approach was previously used by Masaro et al. to model water diffusion in aqueous solutions of PEO with other hydrophilic polymers such as poly(vinyl alcohol).(33, 34) In this case, Eqn. S12 simplifies to:(31)

$$D = D_0 \exp\left[- B\left(\frac{1}{\phi_w} - 1\right)\right] \quad \text{Eqn. S13}$$

where ϕ_w is the volume fraction of water in the solution, and B remains a constant proportional to the minimum local free volume required for water diffusion to occur and to the overall free volume of pure liquid water. Water volume fractions were determined according to Eqn. S9.

The value of B was determined by the best fit of experimental PFG-NMR water self-diffusivities to Eqn. S10 using non-linear least squares regression. For 550 g/mol aqueous PEO solutions at 21 °C, B was determined to have a value of 2.28 (R^2 = 0.994), while for aqueous glycerol solutions at 21 °C, B was determined to have a value of 1.90 (R^2 = 0.996).

MD free volume and diffusion simulations

The Califano method for estimating the accessible FFV relies on first inserting a 0.53 Å radius probe "molecule" at every node of a 3D grid (with 0.5 Å bin widths) cast over the simulation box and checking for overlaps with the VDW radii of atoms in the bin volume.(35) The probe size was chosen to match that used by experimental Positron Annihilation Lifetime Spectroscopy (PALS) in which polymer FFV is estimated using the lifetime of ortho-positroniums which have a 0.53 Å radius. If the node has no overlaps with atoms in the bin volume, it is considered an unoccupied node. A bin will be assigned a total free volume depending on the number of unoccupied nodes it contains. A bin with a single unoccupied node has a free volume of 1/8-th the volume of the probe molecule or 0.0779 Å^3. Two, three, and four unoccupied nodes correspond to free volume elements of 0.1020 Å^3, 0.1143 Å^3, and 0.1173 Å^3, respectively. These volumes are estimated by subtracting the average probe overlap volume from the sum of the probe volumes for the 2, 3, and four unoccupied node cases. If there are five or more unoccupied nodes, the entire bin is considered a free volume of size 0.125 Å^3. We note that that, outside of extreme cases (i.e., using a probe of size 0 Å), most combinations of bin size and probe size yield qualitatively similar values. For every simulated PEO-water composition (from 0 to 50 wt%), we check for probe overlaps with the van der Waals radii of any water or polymer molecule atoms (R_H = 1.2 Å, R_C = 1.7 Å, R_O = 1.52 Å).

We further note that accessible FFV values derived from MD are highly sensitive to the choice of probe size and grid spacing. We demonstrate the effect of probe size by inspecting the FFV-concentration (c.f., Figure S3A) and FFV-diffusivity (c.f., Figure S3B) scaling relationships. Ultimately, while the probe size impacts the magnitude of the FFV, probe size does not fundamentally alter the underlying FFV trends in PEO-water solutions.

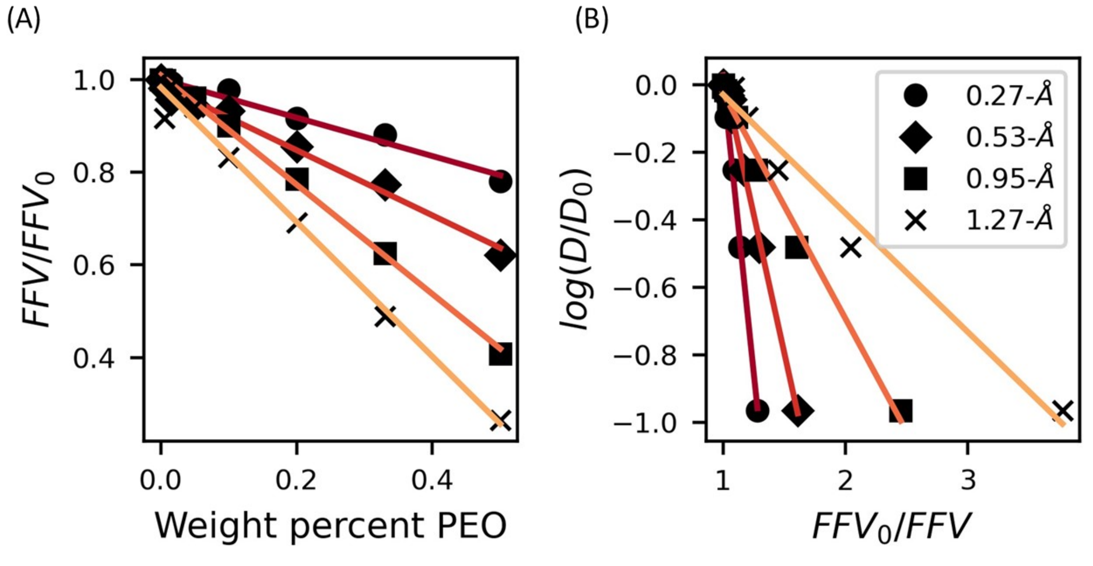

Figure S3. Systematically varying FFV probe size from 0.27 Å to 1.27 Å quantitatively alters the resulting FFV estimate while leaving the underlying relationships with (A) PEO concentration and (B) water self-diffusivity unchanged. Here, we normalize by the pure water fractional free volume FFV_0 to emphasize the consistent exponential relationship between D and $\frac{1}{FFV}$. The 0.27 Å, 0.53 Å, 0.95 Å, and 1.27 Å probe size calculations yield FFV_0 values of 0.332, 0.205, 0.036, and 0.004, respectively.

At dilute PEO concentrations (c<c*), MD-derived water self-diffusivities show near quantitative agreement with the PFG-NMR results, exhibiting less than 20% deviation. Much of this difference may be explained by the 4 °C lower simulated temperature. For instance, NMR studies have previously demonstrated as much as a 15% increase in water self-diffusivity going from 15 to 20°C (36). However, MD simulations give a water self-diffusivity just under half of the PFG-NMR measurement at 50 wt%. This more dramatic discrepancy may stem from imperfections in the ability of the MD model (see Methods) to reproduce accurate equilibrium self-diffusivities at high PEO concentration. Though the PEO parameters previously yielded accurate conformational landscapes (3) and phase behavior in water,(37) it was not validated with equilibrium dynamics in mind.

ODNP measurement of water, PEO, and free TEMPOL

In addition to ODNP experiments on spin-labeled PEO, we performed ODNP experiments on mixtures of water and unlabeled PEO (550 g/mol, 0-90 wt%) with free 4-hydroxy-TEMPO (TEMPOL) (Sigma-Aldrich 176141, used as purchased) Because the free TEMPOL is well dispersed in aqueous solution, results report on the average dynamics of water non-local to the PEO chain end, which may approximate the bulk dynamics reflected in the PFG-NMR experiments. Measurements were performed at 18°C and identical conditions as the PEO-labeled results. We observe systematically higher diffusivities than the PEO-labeled ODNP results, with fairly consistent agreement in magnitude and trend as the PFG-NMR water diffusivities. It should be noted that for these samples, the determination of the saturation factor is more uncertain than the PEO-labeled, case, as we cannot assume a value of 1 due to tethering of the spin label. In these results, we assume a constant value of 0.359, as this is the predicted value for free TEMPOL in pure water. The concentration of PEO may affect the saturation factor by either Heisenberg exchange or ^{14}N relaxation, but a more thorough analysis lies beyond the scope of this paper. Regardless, these data demonstrate a clear slowing effect of the PEO surface on the ODNP-derived diffusivity of water.

The effect of the saturation factor on the PEO-labeled ODNP data may be assumed to be minimal. In a study by Hyde and coworkers, they determined that for spin-labeled doxyl-stearic acid, which has a comparable molecular weight to our spin-labeled PEO, when placed in a lipid bilayer has a value of the nitrogen relaxation that corresponds to a = 0.92.(38) Even if we assume our PEO is more mobile, and take s_{max} = 0.9, we would observe only an ~8% increase in the water diffusivity. Thus it can be reasonably stated that the uncertainty in s_{max} on the PEO-tethered ODNP water diffusivity lies within the ~20% experimental error on the data.

We further note that the effect of the ODNP probe on local water diffusivities is not responsible for the diffusivity trends reported in this study. First, the ODNP probe is relatively small compared to other more hydrophobic probes. Second, ODNP data are compared to a reference value of the small nitroxide spin probe freely dissolved in water. This has been shown to be an adequate control for local effects from interactions between the nitroxide moiety and nearby water molecules. Many careful studies, including this one, have been done which show that the ODNP water diffusion coefficient near spin labels in solution or surfaces report on the water properties modulated by the solutes or surfaces of interest, not the spin label.(39–41) The reason ODNP is able to capture local, surface water dynamics is due to the strength of the extended hydrogen bond network; the influence of a contiguous surface is the largest factor affecting local water dynamics. Our recent work demonstrates significant correlations between ODNP water diffusivities and several MD simulated water structural and dynamic order parameters that are well-established in the literature.(42) See also Figure S8 for correlations between MD and ODNP water diffusivities and tetrahedral water populations in this work. Furthermore, our data is internally consistent by virtue of the ODNP-derived value in PEO solution showing no concentration dependence up to a critical polymer concentration. This indicates that the effect of the ODNP probe is minimal in the report of water diffusion for this study.

Mutual diffusion coefficients

According to Vrentas and Duda following previous work by Bearman, mutual diffusion coefficients in binary solvent-polymer systems can under some circumstances be estimated from solvent and polymer self-diffusion coefficients.(28, 43) If a relationship between the three solvent-solvent, solvent-polymer, and polymer-polymer friction coefficients is assumed, one can write an expression for the solvent-polymer mutual diffusion coefficient, D_{wp}, as: (28, 43)

$$D_{wp} = \frac{D_w x_p + D_p x_w}{RT}\left(\frac{\partial \mu_w}{\partial \ln x_w}\right)_{T,p} \quad \text{Eqn. S14}$$

where D_w and D_p are water and polymer self-diffusion coefficients, respectively [cm^2/s], x_w and x_p are water and polymer mole fractions, respectively, R is the ideal gas constant (8.314 J/mol·K), T is absolute temperature (~293 K for the present study), and μ_w is the chemical potential of water [J/mol]. Eqn. S14 can be rewritten in terms of the activity of water, a_w, and the water volume fraction of the mixture:

$$D_{wp} = (D_w x_p + D_p x_w)\frac{\partial \ln a_w}{\partial \ln x_w} = (D_w x_p + D_p x_w)\frac{\partial \ln a_w \partial \phi_w}{\partial \phi_w \ \partial x_w} x_w \quad \text{Eqn. S15}$$

The water activity partial derivative term can be approximated using Flory-Huggins solution theory:(44, 45)

$$\frac{\partial \ln a_w}{\partial \phi_w} = \frac{1}{\phi_w} - \left(1 - \frac{1}{r}\right) - 2\chi_{wp} + 2\chi_{wp}\phi_w \quad \text{Eqn. S16}$$

where r the number of effective segments in a polymer chain which have approximately the same volume as a solvent molecule and which is assumed to equal the ratio of the molar volumes of the polymer and water ($r = 27$), and χ_{wp} is the Flory-Huggins interaction parameter. Flory-Huggins parameters for PEO-water mixtures were estimated from water activity data from Ninni et al. and are shown in Figure S4.(46)

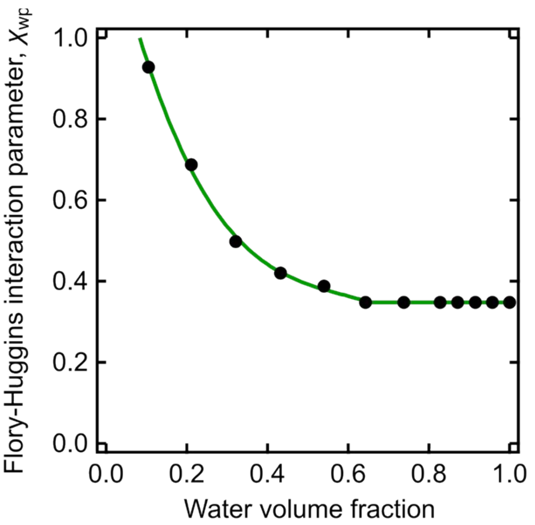

Figure S4. Flory-Huggins interaction parameters for PEO-water solutions as a function of water volume fraction derived from water activity data from Ninni et al.(46)

Water volume fractions can be related to water mole fractions assuming volume additivity by approximating the partial specific volume of water as constant and equal to the specific volume of pure water:

$$\phi_w = \frac{\frac{18.02}{\rho_w}x_w}{\frac{18.02}{\rho_w}x_w + \frac{550}{\rho_p}(1 - x_w)} \qquad \text{Eqn. S17}$$

where ρ_w and ρ_p are mass densities of pure water and pure PEO, respectively [g/cm^3]. The volume fraction derivative term in Eqn. S15 can be obtained from the first derivative of Eqn. S17:

$$\frac{\partial \phi_w}{\partial x_w} = \frac{18.02(550)}{\rho_w \rho_p \left(\frac{18.02}{\rho_w}x_w + \frac{550}{\rho_p}(1 - x_w)\right)} \qquad \text{Eqn. S18}$$

The term $\frac{\partial \ln a_w}{\partial \ln x_w}$ in Eqn. S15 can thus be derived as the product of the expressions in Eqn. S16 and S18 and water mole fraction:

$$\frac{\partial \ln a_w}{\partial \ln x_w} = \left[\frac{1}{\phi_w} - \left(1 - \frac{1}{r}\right) - 2\chi_{wp} + 2\chi_{wp}\phi_w\right]\left[\frac{18.02(550)}{\rho_w \rho_p \left(\frac{18.02}{\rho_w}x_w + \frac{550}{\rho_p}(1 - x_w)\right)}\right]x_w \qquad \text{Eqn. S19}$$

Eqn. S19 represents a "thermodynamic factor" that accounts for a thermodynamic driving force for mass transfer in the presence of a concentration gradient or fluctuations, which is plotted as a function of water volume fraction in Figure S5.(47) An attempt to account for the effects of non-ideal mixing in the thermodynamic factor resulted in negligible qualitative differences from those obtained from the volume additivity assumption, so only the simpler volume additivity approximation is reported here.

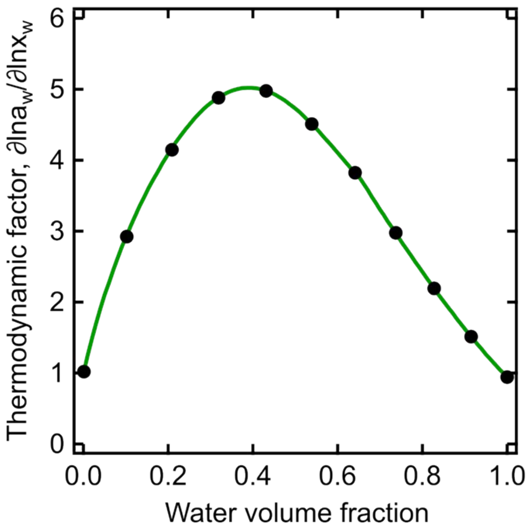

Figure S5. Thermodynamic factors for PEO-water solutions as a function of water volume fraction derived according to Eqn. S19.

By substituting the thermodynamic factor, $\frac{\partial \ln a_w}{\partial \ln x_w}$, into Eqn. S15, one can obtain an expression relating D_{wp} to water and polymer self-diffusion coefficients, D_w and D_p, and the mole fractions of water and polymer in the solution. Water-PEO mutual diffusion coefficients thereby obtained are compared with water and polymer self-diffusion coefficients obtained via PFG-NMR and water-PEO relative diffusion coefficients obtained via spin-labeled PEO ODNP measurements in Figure S6.

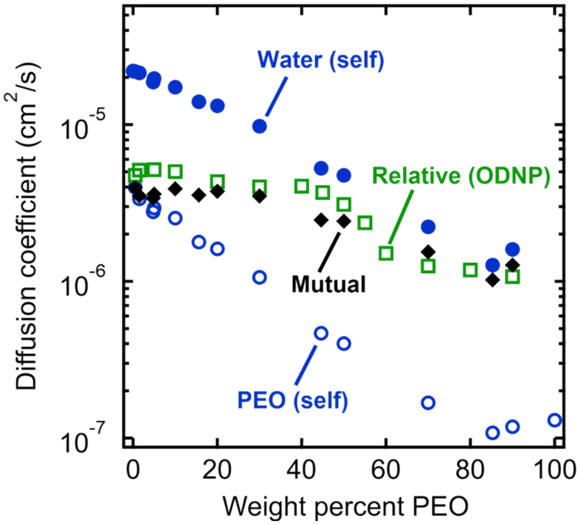

Figure S6. Comparison of water self-diffusion coefficients obtained via PFG-NMR (filled blue circles), PEO self-diffusion coefficients obtained via PFG-NMR (unfilled blue circles), water-PEO relative diffusion coefficients obtained via spin-labeled PEO ODNP (unfilled green squares), and water-PEO mutual diffusion coefficients estimated from Eqn. S15 (filled black diamonds) in PEO-water solutions.

MD simulations of solution structure

Though there are several metrics that report on the tetrahedrality of water in molecular simulations(48–50), many of these metrics can underestimate water coordination near surfaces(48, 49, 51, 52) due to the requirement of 4 neighboring waters. On the other hand, 3-body angle distributions do not require a given water molecule to be 4-coordinated, making them more robust to the geometric constraints introduced at interfaces.

Water's tetrahedral structure is closely related to its capacity to form approximately 4 hydrogen bonds per water molecule. In the present study, we note that both the number of water-water hydrogen bonds per water molecule, $N_{HB,wat-wat}/N_w$, and p_{tet} strongly correlate with the FFV of PEO-water mixtures (Figure S7A-B) and with each other (Figure S7C). The overall decrease in $N_{HB,wat-wat}/N_w$ with increasing concentration in Figure S7 is driven by the waters that directly hydrogen bond with PEO ether oxygens. The remaining hydration waters on the other hand exhibit enhanced tetrahedrality, with the net effect of an overall enhancement of the hydration layer tetrahedral structure.

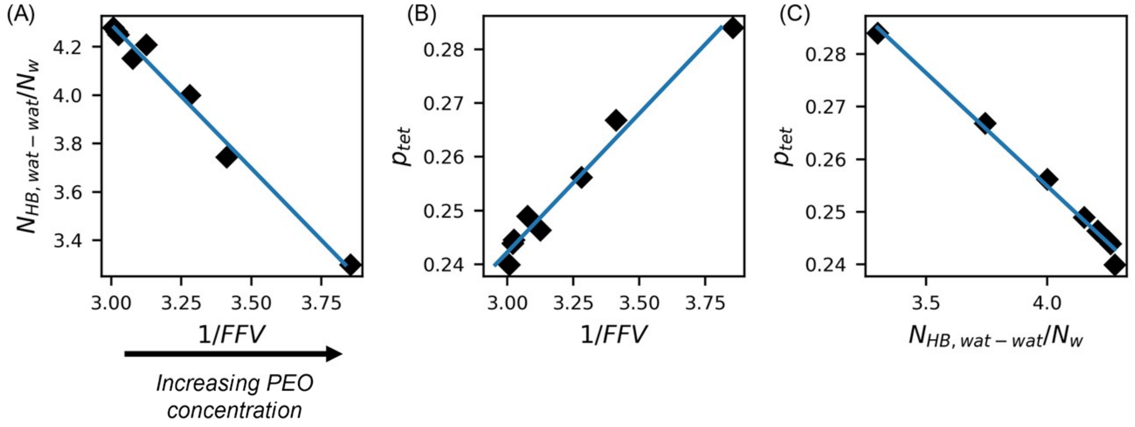

Figure S7. The MD-computed FFV exhibits strong correlations with water structural metrics such as (A) the average number of water-water hydrogen bonds per water molecule, $N_{HB,wat-wat}/N_w$, and (B) the population of tetrahedrally coordinated waters, p_{tet}. (C) p_{tet} correlates strongly with $N_{HB,wat-wat}/N_w$.

Correlations between MD and ODNP water diffusivities and tetrahedral water population

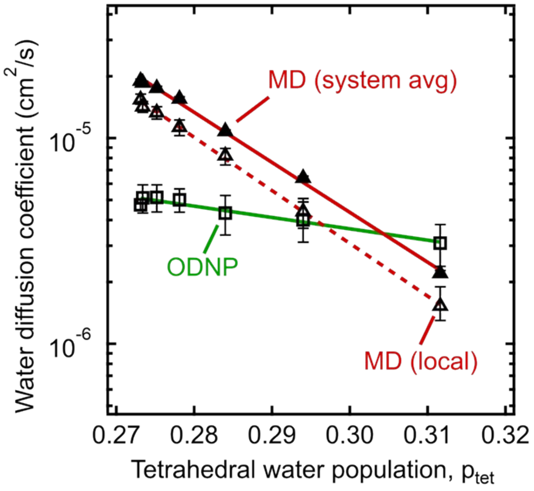

Figure S8. Comparing the correlation between population of tetrahedral waters p_{tet} and three estimates of the relative water diffusivity: system-averaged water self-diffusivity from MD D_{H_2O} (filled triangles), water self-diffusivity for waters within 0.8 nm of the radical oxygen from MD D_{local} (unfilled triangles), and ODNP-derived local water self-diffusivity using spin-labeled PEO D_{ODNP} (unfilled squares). Both MD estimates of water diffusivity show strong correlation with p_{tet} ($R^2 > 0.99$). While the ODNP results show a nominally favorable correlation with p_{tet} ($R^2 = 0.95$), the slope of the relationship is significantly smaller than the former two cases.

ODNP characterization of water dynamics in higher molecular weight PEO solutions

Beyond the measurements of 550 Da studied in this work, we performed ODNP screening measurements of spin-labeled PEO in water at MW = 800, 3000, and 32,000 Da at dilute concentrations. These polymer solutions all show comparable ODNP k_σ water dynamics results (Figure S9). The ODNP parameter k_σ scales with water diffusivity and reports exclusively on fast timescale (100s ps) water motion.(40, 41) We have not performed a more systematic study of different molecular weight PEO at different concentrations using ODNP, nor performed these experiments using PFG-NMR because it was outside the scope of this study. We expect that the same general trend and mechanisms apply to higher molecular weights at the same nominal water contents.

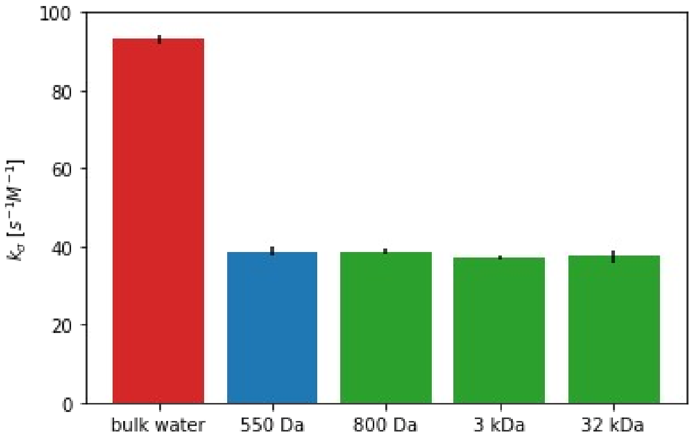

Figure S9. Comparing ODNP k_σ water dynamics near dilute, spin-labeled PEO at 550 Da (molecular weight used in this study) with three higher molecular weights: 800 Da, 3 kDa, and 32 kDa. The value for "bulk water" represents a system with 200 µM free TEMPOL in pure water. No significant difference is found between the water dynamics near PEO of varying molecular weight. Error bars represent one standard deviation of three experimental repeats.

Tabulated water and PEO diffusion coefficients

Table S1. PFG-NMR time-averaged self-diffusion coefficients for aqueous PEO (550 g/mol) solutions at 21 °C.

Water weight percent	PEO weight percent	Water time-averaged self-diffusivity [10^{-6} cm^2/s]	PEO time-averaged self-diffusivity [cm^2/s]
100.0	0.0	22.0	3.98
99.5	0.5	21.8	3.98
98.5	1.5	21.4	3.36
95.2	4.8	18.7	2.78
95.0	5.0	19.7	2.94
90.0	10.0	17.3	2.53
84.4	15.6	14.0	1.78
80.0	20.0	13.2	1.61
70.0	30.0	9.76	1.06
55.4	44.6	5.26	0.47
50.0	50.0	4.74	0.40
30.0	70.0	2.23	0.17
14.7	85.3	1.27	0.11
10.0	90.0	1.60	0.12
0.0	100.0	-	0.13

Table S2. PFG-NMR time-averaged self-diffusion coefficients for aqueous glycerol solutions at 21 °C.

Water weight percent	Glycerol weight percent	Water time-averaged self-diffusivity [10^{-6} cm^2/s]	Glycerol time-averaged self-diffusivity [cm^2/s]
100.0	0.0	22.0	-
78.8	21.2	14.1	5.39
63.8	36.2	9.15	3.40
43.9	56.1	3.92	1.43
31.3	68.7	1.95	0.66
22.7	77.3	0.96	0.34
16.4	83.6	-	0.17
0.0	100.0	-	0.013

Table S3. ODNP relative water diffusion coefficients for aqueous PEO (550 g/mol) solutions at 18 °C.

Water weight percent	PEO weight percent	Water diffusivity, PEO-tethered spin labels [10^{-6} cm^2/s]	Water diffusivity, free TEMPOL spin labels [10^{-6} cm^2/s]
100	0	-	18.1 ± 12.9
99.5	0.5	4.74 ± 0.28	-
98.5	1.5	5.12 ± 0.80	10.1 ± 0.93
95	5	5.15 ± 0.78	11.5 ± 2.80
90	10	5.01 ± 0.65	12.3 ± 0.26
80	20	4.32 ± 0.94	8.53 ± 1.30
70	30	4.01 ± 0.89	9.49 ± 3.59
60	40	4.05 ± 0.64	-
55	45	3.69 ± 0.73	-
50	50	3.09 ± 0.71	4.81 ± 1.24
45	55	2.36 ± 1.11	-
40	60	1.51 ± 0.18	-
30	70	1.25 ± 0.29	4.42 ± 0.74
20	80	1.18 ± 0.48	-
10	90	1.07 ± 0.83	1.52 ± 0.10

Table S4. MD equilibrium water self-diffusion coefficients for aqueous PEO (550 g/mol) solutions at 17 °C.

Water weight percent	PEO weight percent	Local water self-diffusivity [10^{-6} cm^2/s]	System-averaged water self-diffusivity [cm^2/s]
100	0	$16.3\ ^{+\ 0.76}_{-\ 0.96}$	$19.3\ ^{+\ 0.60}_{-\ 0.69}$
99.5	0.5	$15.4\ ^{+\ 0.96}_{-\ 0.75}$	$19.0\ ^{+\ 0.40}_{-\ 0.34}$
98.5	1.5	$14.3\ ^{+\ 0.84}_{-\ 0.86}$	$18.6\ ^{+\ 0.31}_{-\ 0.32}$
95	5	$13.3\ ^{+\ 0.92}_{-\ 0.94}$	$17.5\ ^{+\ 0.39}_{-\ 0.42}$
90	10	$11.3\ ^{+\ 0.95}_{-\ 1.02}$	$15.5\ ^{+\ 0.40}_{-\ 0.48}$
80	20	$8.21\ ^{+\ 0.69}_{-\ 0.82}$	$10.8\ ^{+\ 0.16}_{-\ 0.15}$
67	33	$4.40\ ^{+\ 0.69}_{-\ 0.75}$	$6.40\ ^{+\ 0.14}_{-\ 0.13}$
50	50	$1.53\ ^{+\ 0.36}_{-\ 0.23}$	$2.20\ ^{+\ 0.07}_{-\ 0.06}$

Supporting Information references

1. P.-G. de Gennes, *Scaling Concepts in Polymer Physics* (Cornell University Press, 1979).

2. M. Doi, S. F. Edwards, *The Theory of Polymer Dynamics* (Clarendon Press, 1988).

3. N. Sherck, *et al.*, End-to-End Distance Probability Distributions of Dilute Poly(ethylene oxide) in Aqueous Solution. *J. Am. Chem. Soc.* **142**, 19631–19641 (2020).

4. W. C. Smith, *et al.*, Determining critical overlap concentration of polyethylene oxide to support excipient safety assessment of opioid products. *Int. J. Pharm.* **632**, 122557 (2023).

5. D. C. Vadillo, W. Mathues, C. Clasen, Microsecond relaxation processes in shear and extensional flows of weakly elastic polymer solutions. *Rheol. Acta* **51**, 755–769 (2012).

6. W. W. Graessley, Polymer chain dimensions and the dependence of viscoelastic properties on concentration, molecular weight and solvent power. *Polymer* **21**, 258–262 (1980).

7. G. M. Harrison, J. Remmelgas, L. G. Leal, The dynamics of ultradilute polymer solutions in transient flow: Comparison of dumbbell-based theory and experiment. *J. Rheol.* **42**, 1039–1058 (1998).

8. L. Ansaloni, Minelli, Matteo, Baschetti, M. Giacinti, G. C. Sarti, Effect of relative humidity and temperature on gas transport in Matrimid(R): Experimental study and modeling. *J. Membr. Sci.* **471**, 392–401 (2014).

9. J. D. Moon, *et al.*, Water Vapor Sorption, Diffusion, and Dilation in Polybenzimidazoles. *Macromolecules* **51**, 7197–7208 (2018).

10. J. D. Moon, *et al.*, Modeling water diffusion in polybenzimidazole membranes using partial immobilization and free volume theory. *Polymer* **189**, 122170 (2020).

11. W. M. Lee, Selection of barrier materials from molecular structure. *Polym. Eng. Sci.* **20**, 65–69 (1980).

12. N. R. Horn, A critical review of free volume and occupied volume calculation methods. *J. Membr. Sci.* **518**, 289–294 (2016).

13. A. Bondi, van der Waals Volumes and Radii. *J. Phys. Chem.* **68**, 441–451 (1964).

14. J. Y. Park, D. R. Paul, Correlation and prediction of gas permeability in glassy polymer membrane materials via a modified free volume based group contribution method. *J. Membr. Sci.* **125**, 23–39 (1997).

15. A. X. Wu, *et al.*, Revisiting group contribution theory for estimating fractional free volume of microporous polymer membranes. *J. Membr. Sci.*, 119526 (2021).

16. J. T. Edward, Molecular volumes and the Stokes-Einstein equation. *J. Chem. Educ.* **47**, 261 (1970).

17. I. Moriguchi, Y. Kanada, K. Komatsu, van der Waals Volume and the Related Parameters for Hydrophobicity in Structure-Activity Studies. *Chem. Pharm. Bull. (Tokyo)* **24**, 1799–1806 (1976).

18. J. R. Scherer, B. A. Bolton, Water in polymer membranes. 5. On the existence of pores and voids. *J. Phys. Chem.* **89**, 3535–3540 (1985).

19. J. R. Scherer, The partial molar volume of water in biological membranes. *Proc. Natl. Acad. Sci.* **84**, 7938–7942 (1987).

20. R. F. W. Bader, M. T. Carroll, J. R. Cheeseman, C. Chang, Properties of atoms in molecules: atomic volumes. *J. Am. Chem. Soc.* **109**, 7968–7979 (1987).

21. S. Trivedi, C. Bhanot, S. Pandey, Densities of {poly(ethylene glycol)+water} over the temperature range (283.15 to 363.15)K. *J. Chem. Thermodyn.* **42**, 1367–1371 (2010).

22. E. Ayranci, M. Sahin, Interactions of polyethylene glycols with water studied by measurements of density and sound velocity. *J. Chem. Thermodyn.* **40**, 1200–1207 (2008).

23. A. Volk, C. J. Kähler, Density model for aqueous glycerol solutions. *Exp. Fluids* **59**, 75 (2018).

24. C. Bhanot, S. Trivedi, A. Gupta, S. Pandey, S. Pandey, Dynamic viscosity versus probe-reported microviscosity of aqueous mixtures of poly(ethylene glycol). *J. Chem. Thermodyn.* **45**, 137–144 (2012).

25. M. L. Sheely, Glycerol Viscosity Tables. *Ind. Eng. Chem.* **24**, 1060–1064 (1932).

26. J. S. Mackie, P. Meares, The diffusion of electrolytes in a cation-exchange resin membrane I. Theoretical. *Proc. R. Soc. Lond. Ser. Math. Phys. Sci.* **232**, 498–509 (1955).

27. M. H. Cohen, D. Turnbull, Molecular Transport in Liquids and Glasses. *J. Chem. Phys.* **31**, 1164–1169 (1959).

28. J. S. Vrentas, J. L. Duda, Diffusion in polymer—solvent systems. I. Reexamination of the free-volume theory. *J. Polym. Sci. Polym. Phys. Ed.* **15**, 403–416 (1977).

29. J. S. Vrentas, J. L. Duda, Diffusion in polymer–solvent systems. II. A predictive theory for the dependence of diffusion coefficients on temperature, concentration, and molecular weight. *J. Polym. Sci. Polym. Phys. Ed.* **15**, 417–439 (1977).

30. H. Yasuda, C. E. Lamaze, A. Peterlin, Diffusive and hydraulic permeabilities of water in water-swollen polymer membranes. *J. Polym. Sci. Part -2 Polym. Phys.* **9**, 1117–1131 (1971).

31. H. Yasuda, C. E. Lamaze, L. D. Ikenberry, Permeability of solutes through hydrated polymer membranes. Part I. Diffusion of sodium chloride. *Makromol. Chem.* **118**, 19–35 (1968).

32. H. Yasuda, A. Peterlin, C. K. Colton, K. A. Smith, E. W. Merrill, Permeability of solutes through hydrated polymer membranes. Part III. Theoretical background for the selectivity of dialysis membranes. *Makromol. Chem.* **126**, 177–186 (1969).

33. L. Masaro, M. Ousalem, W. E. Baille, D. Lessard, X. X. Zhu, Self-Diffusion Studies of Water and Poly(ethylene glycol) in Solutions and Gels of Selected Hydrophilic Polymers. *Macromolecules* **32**, 4375–4382 (1999).

34. L. Masaro, X. X. Zhu, P. M. Macdonald, Study of the self-diffusion of poly(ethylene glycol)s in poly(vinyl alcohol) aqueous systems. *J. Polym. Sci. Part B Polym. Phys.* **37**, 2396–2403 (1999).

35. D. Racko, R. Chelli, G. Cardini, J. Bartos, S. Califano, Insights into positron annihilation lifetime spectroscopy by molecular dynamics simulations: Free-volume calculations for liquid and glassy glycerol. *Eur. Phys. J. D* **32**, 289–297 (2005).

36. P. S. Tofts, *et al.*, Test liquids for quantitative MRI measurements of self-diffusion coefficient in vivo. *Magn. Reson. Med.* **43**, 368–374 (2000).

37. N. Sherck, *et al.*, Molecularly Informed Field Theories from Bottom-up Coarse-Graining. *ACS Macro Lett.* **10**, 576–583 (2021).

38. C. A. Popp, J. S. Hyde, Electron-electron double resonance and saturation-recovery studies of nitroxide electron and nuclear spin-lattice relaxation times and Heisenberg exchange rates: lateral diffusion in dimyristoyl phosphatidylcholine. *Proc. Natl. Acad. Sci. U. S. A.* **79**, 2559–2563 (1982).

39. C.-Y. Cheng, J. Varkey, M. R. Ambroso, R. Langen, S. Han, Hydration dynamics as an intrinsic ruler for refining protein structure at lipid membrane interfaces. *Proc. Natl. Acad. Sci.* **110**, 16838–16843 (2013).

40. R. Barnes, *et al.*, Spatially Heterogeneous Surface Water Diffusivity around Structured Protein Surfaces at Equilibrium. *J. Am. Chem. Soc.* **139**, 17890–17901 (2017).

41. J. M. Franck, Y. Ding, K. Stone, P. Z. Qin, S. Han, Anomalously Rapid Hydration Water Diffusion Dynamics Near DNA Surfaces. *J. Am. Chem. Soc.* **137**, 12013–12023 (2015).

42. D. C. Robinson Brown, *et al.*, Relationships between Molecular Structural Order Parameters and Equilibrium Water Dynamics in Aqueous Mixtures. *J. Phys. Chem. B* (2023) https:/doi.org/10.1021/acs.jpcb.3c00826 (May 16, 2023).

43. R. J. Bearman, On the molecular basis of some current theories of diffusion. *J. Phys. Chem.* **65**, 1961–1968 (1961).

44. P. Flory J., Thermodynamics of High Polymer Solutions. *Journal of Chemical Physics* **10**, 51–61 (1942).

45. M. L. Huggins, Solutions of Long Chain Compounds. *J. Chem. Phys.* **9**, 440–440 (1941).

46. L. Ninni, M. S. Camargo, A. J. A. Meirelles, Water activity in poly(ethylene glycol) aqueous solutions. *Thermochim. Acta* **328**, 169–176 (1999).

47. F. Doghieri, G. C. Sarti, Solubility, Diffusivity, and Mobility of n-Pentane and Ethanol in Poly(1-trimethylsilyl-1-propyne). *J. Polym. Sci. Part B Polym. Phys.* **35**, 2245–2258 (1997).

48. J. I. Monroe, M. S. Shell, Decoding signatures of structure, bulk thermodynamics, and solvation in three-body angle distributions of rigid water models. *J. Chem. Phys.* **151**, 094501 (2019).

49. A. Chaimovich, M. S. Shell, Tetrahedrality and structural order for hydrophobic interactions in a coarse-grained water model. *Phys. Rev. E* **89** (2014).

50. J. R. Errington, P. G. Debenedetti, Relationship between structural order and the anomalies of liquid water. *Nature* **409**, 318–321 (2001).

51. P. Stock, *et al.*, Unraveling Hydrophobic Interactions at the Molecular Scale Using Force Spectroscopy and Molecular Dynamics Simulations. *ACS Nano* **11**, 2586–2597 (2017).

52. J. Monroe, *et al.*, Water Structure and Properties at Hydrophilic and Hydrophobic Surfaces. *Annu. Rev. Chem. Biomol. Eng.* **11**, 523–557 (2020).
